# Supplementary material for: Comprehensive multi-metric analysis of user experience and performance in adaptive and non-adaptive lower-limb exoskeletons
Source: PLoS One. 2025 Jan 9;20(1):e0313593. doi: 10.1371/journal.pone.0313593 (PMC11717227; doi:10.1371/journal.pone.0313593)
Supplement: S1 Text — (PDF) [file pone.0313593.s002.pdf]

แบบเสนอโครงการวิจัยเพื่อรับการพิจารณาจากคณะกรรมการจริยธรรมการวิจัยในคน
คณะแพทยศาสตร์โรงพยาบาลรามาธิบดี มหาวิทยาลัยมหิดล (ฉบับเต็ม)

1. ชื่อโครงการ

(ไทย) การพัฒนาระบบโครงกระดูกภายนอกเพื่อช่วยเหลือและเสริมสร้างการเคลื่อนที่สำหรับผู้ที่มีปัญหาทางด้านการเคลื่อนไหว

(อังกฤษ) The Development of an Exoskeleton System for Assisting and Enhancing Patients' Mobility

2. ชื่อหัวหน้าโครงการวิจัย

ชื่อหัวหน้าโครงการวิจัยทางภาควิชาออร์โธปิดิกส์ คณะแพทยศาสตร์ โรงพยาบาลรามาธิบดี

(ไทย) นางสาวธันยพร ปะทะธง

(อังกฤษ) Tanyaporn Patathong

คุณวุฒิ วท.บ. (กายภาพบำบัด)

สถานที่ทำงาน ภาควิชาออร์โธปิดิกส์ คณะแพทยศาสตร์โรงพยาบาลรามาธิบดี ม.มหิดล

สถานที่ที่สามารถติดต่อได้ ภาควิชาออร์โธปิดิกส์ คณะแพทยศาสตร์โรงพยาบาลรามาธิบดี ม.มหิดล

โทรศัพท์ 081-491-6382

ชื่อหัวหน้าโครงการวิจัยร่วมทางสำนักวิชาวิทยาศาสตร์ และเทคโนโลยีสารสนเทศ (IST) สถาบันวิทยสิริเมธี (VISTEC)

(ไทย) ศาสตราจารย์ ดร.ปรเมษฐ์ มนูญพงศ์

(อังกฤษ) Prof. Dr. Poramate Manoonpong

คุณวุฒิ ศาสตราจารย์

สถานที่ทำงาน สำนักวิชาวิทยาศาสตร์และเทคโนโลยีสารสนเทศ (IST) ของสถาบันวิทยสิริเมธี

สถานที่ที่สามารถติดต่อได้ สำนักวิชาวิทยาศาสตร์และเทคโนโลยีสารสนเทศ (IST) ของสถาบันวิทยสิริเมธี (VISTEC) 555 ม. 1 ต. ป่ายุบใน อ. วังจันทร์ จ. ระยอง 21210

โทรศัพท์ 095-709-2635

3. ชื่อผู้ร่วมการวิจัย

3.1 (ไทย) รองศาสตราจารย์ ดร. แพทย์หญิง ภัทรวัณย์ วรธนารัตน์

(อังกฤษ) Assoc.Prof.Dr. Patarawan Woratanarat, M.D., Ph.D.

คุณวุฒิ รองศาสตราจารย์

สถานที่ทำงาน ภาควิชาออร์โธปิดิกส์คณะแพทยศาสตร์โรงพยาบาลรามาธิบดี ม.มหิดล

สถานที่ที่สามารถติดต่อได้ ภาควิชาออร์โธปิดิกส์คณะแพทยศาสตร์โรงพยาบาลรามาธิบดี ม.มหิดล

โทรศัพท์ 02-201-1589

3.2 (ไทย) ผู้ช่วยศาสตราจารย์ แพทย์หญิงชนิกา อังสนันท์สุข

(อังกฤษ) Assist.Prof.Dr.Chanika Angsanunsukh, M.D.

คุณวุฒิ ผู้ช่วยศาสตราจารย์

สถานที่ทำงาน ภาควิชาออร์โธปิดิกส์ คณะแพทยศาสตร์โรงพยาบาลรามาธิบดี ม.มหิดล

สถานที่ที่สามารถติดต่อได้ ภาควิชาออร์โธปิดิกส์ คณะแพทยศาสตร์โรงพยาบาลรามาธิบดี ม.มหิดล

โทรศัพท์ 02-201-1589

3.3 (ไทย) ผู้ช่วยศาสตราจารย์ นายแพทย์ นรเทพ กุลโชติ

(อังกฤษ) Asst. Prof. Dr. Noratep Kulachote, M.D.

คุณวุฒิ ผู้ช่วยศาสตราจารย์

สถานที่ทำงาน ภาควิชาออร์โธปิดิกส์ คณะแพทยศาสตร์โรงพยาบาลรามาธิบดี ม.มหิดล

สถานที่ที่สามารถติดต่อได้ ภาควิชาออร์โธปิดิกส์ คณะแพทยศาสตร์โรงพยาบาลรามาธิบดี ม.มหิดล

โทรศัพท์ 02-201-1589

3.4 (ไทย) นางสาวกรองแก้ว แกล้วกสิกรรม

(อังกฤษ) Krongkaew Klaewkasikam

คุณวุฒิ วท.บ. (กายภาพบำบัด)

สถานที่ทำงาน ภาควิชาออร์โธปิดิกส์ คณะแพทยศาสตร์โรงพยาบาลรามาธิบดี ม.มหิดล

สถานที่ที่สามารถติดต่อได้ ภาควิชาออร์โธปิดิกส์ คณะแพทยศาสตร์โรงพยาบาลรามาธิบดี ม.มหิดล

โทรศัพท์ 02-201-0412

3.5 (ไทย) นาย ชายชาญ อรรควุฒิวาณิชย์

(อังกฤษ) Chaicharn Akkawutvanich

คุณวุฒิ วท.ม. สาขาวิชาวิศกรรมชีวการแพทย์

สถานที่ทำงาน สำนักวิชาวิทยาศาสตร์และเทคโนโลยีสารสนเทศ (IST) ของสถาบันวิทยสิริเมธี

สถานที่ที่สามารถติดต่อได้ สำนักวิชาวิทยาศาสตร์และเทคโนโลยีสารสนเทศ (IST) ของสถาบันวิทยสิริเมธี (VISTEC) 555 ม. 1 ต. ป่ายุบใน อ. วังจันทร์ จ. ระยอง 21210

โทรศัพท์ 081-436-8333

4. ระบุชื่อแพทย์/ผู้ที่จะดูแลอาสาสมัคร (ผู้ยินยอมตนให้ทำวิจัย)และติดต่อได้ 24 ชั่วโมง อย่างน้อย 2 คน

4.1 นางสาวธันยพร ปะทะธง

ที่อยู่(ที่ทำงาน) ภาควิชาออร์โธปิดิกส์ คณะแพทยศาสตร์โรงพยาบาลรามาธิบดี ม.มหิดล

โทรศัพท์ 081-491-6382

4.2 ผู้ช่วยศาสตราจารย์ แพทย์หญิงชนิกา อังสนันท์สุข

ที่อยู่(ที่ทำงาน) ภาควิชาออร์โธปิดิกส์ คณะแพทยศาสตร์โรงพยาบาลรามาธิบดี ม.มหิดล

โทรศัพท์ 02-201-1589

5. หลักการและเหตุผลใส่ reference แบบ in-text-citation (แบบเลขยกกำลัง)

การเดินเป็นพื้นฐานที่สำคัญของการเคลื่อนไหวร่างกายเพื่อประกอบกิจกรรมต่างๆ โดยในปี พ.ศ.2560 ประเทศไทยมีรายงานจำนวนประชากรผู้พิการทางด้านการเคลื่อนไหวจำนวน 878,888 คน[1] ความพิการทางด้านการเคลื่อนไหวอาจส่งผลต่อทั้งสภาพร่างกายและจิตใจของผู้พิการ โดยเฉพาะการกำจัดทางด้านการเคลื่อนไหวซึ่งมีความเสี่ยงที่ทำให้เกิดอาการแทรกซ้อนต่างๆ เช่น แผลกดทับ(pressure lucers), กล้ามเนื้อฝ่อลีบ(muscle atrophy), ข้อต่อยึดติด (joint stiffness), และโรคกระดูกพรุน(osteoporosis)[2] โดยหนึ่งในวิธีที่จะช่วยฟื้นฟูความสามารถทางร่างกายและจิตใจของผู้พิการ คือการพัฒนาเครื่องมือที่มีประสิทธิภาพในการฟื้นฟูด้านการเคลื่อนไหว ให้ผู้พิการสามารถกลับมาดำรงชีวิตร่วมกับผู้อื่นในสังคมได้

ในปัจจุบันมีอุปกรณ์เสริมความสามารถในการฟื้นฟูและปรับปรุงการเคลื่อนไหวร่างกายในผู้พิการ เช่น โครงกระดูกภายนอกสำหรับรยางค์ล่าง (Lower Exoskeleton) ที่ถูกพัฒนามาเพื่อช่วยผู้ใช้การเคลื่อนที่นอกจากนั้น อุปกรณ์ดังกล่าวยังได้นำมาใช้ในงานเวชศาสตร์ฟื้นฟูอีกด้วย[3] โดยทั่วไป ระบบโครงกระดูกภายนอก (Exoskeleton) ที่มีอยู่ในท้องตลาด ถูกนำไปใช้ในหลายจุดประสงค์ ทั้งทางด้านการทหาร, ทางการแพทย์, หรือใช้งานตามบ้าน จุดประสงค์หลักของการใช้งานทางการแพทย์และการใช้งานตามบ้านนั้นเพื่อการฟื้นฟูการยืน การนั่ง และการเดินของผู้ใช้ซึ่งจะช่วยการทำงานทางสรีรวิทยาของร่างกาย[4-9]

ระบบควบคุมExoskeleton ที่มีอยู่โดยส่วนใหญ่อยู่บนพื้นฐานของเทคนิคการกำหนดเส้นทางการเคลื่อนที่ของข้อต่อปราศจากการควบคุมแบบเรียลไทม์และการเรียนรู้อย่างอัตโนมัติ ดังนั้นทำให้ไม่เกิดการทำงานแบบปรับตัวและเปลี่ยนแปลงไปตามปฏิสัมพันธ์ระหว่างผู้ใช้กับอุปกรณ์ได้ โดยปฏิสัมพันธ์นี้มีความสำคัญเพื่อทำให้เกิดการเดินอย่างเป็นธรรมชาติและหลากหลายรูปแบบในสิ่งแวดล้อมที่แตกต่างกันออกไป เช่น การเดินบนพื้นระดับ การเดินขึ้นลงบันได การเดินบนพื้นต่างระดับ นอกจากนี้ ระบบควบคุมโครงกระดูกฯ แบบดั้งเดิมนั้น ต้องใช้การปรับตัวแปรควบคุมให้เหมาะสมด้วยมือหรือแบบออฟไลน์ ทำให้ระบบต้องใช้เวลานานในการปรับตัวให้เข้ากับผู้ใช้แต่ละคนจากสาเหตุที่กล่าวมาข้างต้นจึงเกิดเป็นโครงการส่วนที่ 1 มีวัตถุประสงค์ในการพัฒนาระบบ Exoskeleton เพื่อพัฒนาเพื่อระบบช่วยเหลือและเสริมสร้างการเคลื่อนที่สำหรับผู้ใช้ให้มีความสามารถในการทำงานแบบเรียลไทม์และมีปฏิสัมพันธ์ระหว่างอุปกรณ์กับผู้ใช้เพื่อฟื้นฟูการเดินให้เป็นไปอย่างธรรมชาติ

โครงการส่วนที่2 มีวัตถุประสงค์เพื่อนำเอาระบบ Exoskeleton ที่ได้รับการพัฒนาแล้ว นำเอามาใช้จริงกับกลุ่มอาสาสมัครสุขภาพดี โดยนำเอาระบบการเดินของ Exoskeleton มาประสานกับการตรวจวิเคราะห์การเดิน gait

analysis เพื่อทดสอบประสิทธิภาพของระบบว่าสามารถนำมาใช้ได้จริง และการเคลื่อนไหวเป็นไปอย่างธรรมชาติ และถูกต้องตามวงจรการเดิน (gait cycle) หรือไม่ซึ่งวิจัยนี้จะเป็นประโยชน์ในการพัฒนาอุปกรณ์ทางด้านเวชศาสตร์ฟื้นฟูต่อไป

6.วัตถุประสงค์ของโครงการวิจัย

โครงการวิจัยนี้มีวัตถุประสงค์ที่จะพัฒนาระบบอัจฉริยะของโครงกระดูกภายนอกเพื่อช่วยเหลือและเสริมสร้างการเคลื่อนที่ และศึกษาผลการทำงานของระบบอัจฉริยะกับระบบปฏิบัติการเคลื่อนไหวพื้นฐานของโครงกระดูกภายนอกเพื่อเปรียบเทียบประสิทธิภาพการทำงานในกลุ่มอาสาสมัคร ด้วยอุปกรณ์ของห้องวิเคราะห์การเดิน ประกอบด้วยเครื่องวิเคราะห์การเดิน (3D Motion Gait Analysis), เครื่องตรวจวัดสัญญาณการทำงานของกล้ามเนื้อ (Electromyography) และเครื่องตรวจวัดความสามารถในการใช้ออกซิเจน (Oxygen Consumption) ดังนี้

6.1 วัตถุประสงค์หลัก

5.1.1 เพื่อศึกษาหาค่าเฉลี่ยการเคลื่อนไหวของข้อต่อ หรือ คิเนเมติกส์ (Kinematics) ขณะเดิน บริเวณข้อสะโพก ข้อเข่า และข้อเท้าของอาสาสมัครขณะสวมใส่ชุดโครงกระดูกภายนอก

6.2 วัตถุประสงค์รอง

6.2.1 เพื่อศึกษาหาค่าจลศาสตร์ของข้อต่อ (Kinetics) ขณะเดิน บริเวณข้อสะโพก ข้อเข่า และข้อเท้า ของอาสาสมัครขณะสวมใส่ชุดโครงกระดูกภายนอก

6.2.2 เพื่อศึกษาหาค่าตัวแปรของการเดิน Temporal-spatio(Gait parameters) ขณะเดินของอาสาสมัครขณะสวมใส่ชุดโครงกระดูกภายนอก

6.2.3 เพื่อศึกษาหาค่าการทำงานของกล้ามเนื้อขา (Muscle activity) ขณะเดินของอาสาสมัครขณะสวมใส่ชุดโครงกระดูกภายนอก

6.2.4 เพื่อศึกษาหาค่าการใช้ออกซิเจน (Oxygen consumption) ขณะเดินของอาสาสมัครขณะสวมใส่ชุดโครงกระดูกภายนอก

6.2.5 เพื่อศึกษาค่าการลงน้ำหนักของเท้าทั้งสองข้างขณะเดิน (Dynamic foot pressure) ของอาสาสมัครขณะสวมใส่ชุดโครงกระดูกภายนอก

6.2.6 เพื่อทดสอบประสิทธิภาพของระบบการควบคุม โดยเปรียบเทียบระหว่างระบบอัจฉริยะและระบบพื้นฐานของชุดโครงกระดูกภายนอก

6.2.7 เพื่อศึกษาหาค่าความสะดวกสบายในขณะเดินและสวมใส่ชุดโครงกระดูกภายนอก

7. วิธีวิจัยและแบบแผนการวิจัย ตารางการทำวิจัย

7.1 รูปแบบการวิจัย (Study design)

2x2 Cross-over design

7.2 สถานที่วิจัย

ห้องปฏิบัติการวิเคราะห์การเดิน(Gait Analysis Laboratory)ภาควิชาออร์โธปิดิกส์ คณะแพทยศาสตร์ โรงพยาบาลรามาธิบดี มหาวิทยาลัยมหิดล

7.3 การดำเนินงานวิจัย

โครงการวิจัยนี้ เป็นโครงการร่วมระหว่าง ภาควิชาออร์โธปิดิกส์ คณะแพทยศาสตร์โรงพยาบาลรามาธิบดี มหาวิทยาลัยมหิดลและทางสำนักวิชาวิทยาศาสตร์และเทคโนโลยีสารสนเทศ (IST) ของสถาบันวิทยสิริเมธี (VISTEC) เพื่อศึกษาผลของระบบควบคุมอัจฉริยะของโครงกระดูกภายนอก โดยมีแผนการดำเนินการแบ่งออกเป็น 2ส่วน (รูปที่ 1) ดังนี้

7.3.1 ส่วนที่ 1 (Phase 1) การพัฒนาระบบอัจฉริยะ (Intelligent mode) ของ Exoskeleton ซึ่งจะดำเนินการโดยคณะทีมวิจัยจากสถาบันวิทยสิริเมธี (VISTEC) โดย Phase 1 นี้ จะมีการพัฒนาโมดูลควบคุมต่าง ๆ ดัง**รูปที่ 1** ได้แก่

- ส่วนที่ 1 พัฒนาโมดูลที่ปรับตัวได้ตามผลค่าความต่างของมุม (Error-based adaptation)
- ส่วนที่ 2 พัฒนาโมดูลสร้างการเคลื่อนที่จากพื้นฐานของ Central Pattern Generator (CPG-based generator)
- ส่วนที่ 3 พัฒนาโมดูลสร้างรูปแบบการเคลื่อนที่ให้เหมาะสมกับแต่ละผู้ใช้ (Pattern generation)
  หลังจากพัฒนาระบบควบคุมโครงกระดูกทั้งสามโมดูลแล้ว ทีมวิจัยจากสถาบัน VISTEC จะนำเอาระบบ Exoskeleton ที่พัฒนาแล้ว มาทดสอบกับกลุ่มอาสาสมัครใน Phase 2 ต่อไป

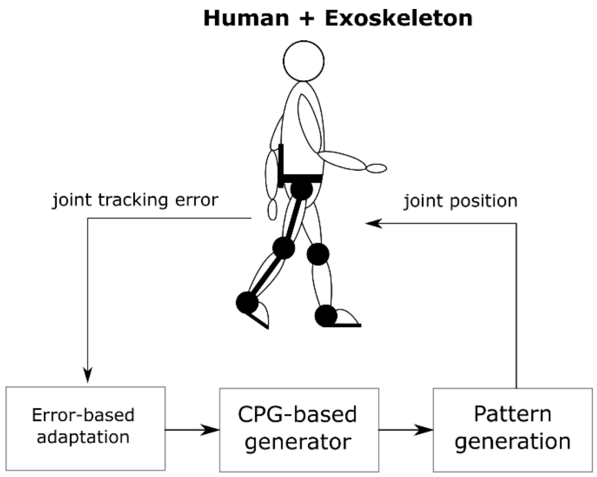

รูปที่ 1 การพัฒนาระบบ Exoskeleton ของทีมวิจัย VISTEC

7.3.2 ส่วนที่ 2 (Phase 2) การนำระบบ Exoskeleton ที่พัฒนาแล้วมาทดลองกับกลุ่มอาสาสมัคร ณ ห้องปฏิบัติการวิเคราะห์การเดิน (Gait Analysis Laboratory) โรงพยาบาลรามาธิบดี มหาวิทยาลัยมหิดล (**รูปที่ 2**) โดยจะมีการทดลอง โดยศึกษาผลการทำงานของระบบ Exoskeleton ที่ได้รับการพัฒนาระบบอัจฉริยะ (Intelligent mode) เพื่อการช่วยเหลือ (Assistive purpose) เปรียบเทียบกับระบบการควบคุมพื้นฐาน (Default mode) ของชุดโครงกระดูกภายนอกในกลุ่มอาสาสมัครสุขภาพดี โดยจะมีการวิเคราะห์การเดิน, การทำงานของกล้ามเนื้อ, การใช้ออกซิเจนของร่างกาย และผลของแรงกดเท้า ของอาสาสมัครขณะไม่สวมใส่ และสวมใส่ Exoskeleton

รูปแบบการทำวิจัยถูกสรุปไว้ใน**รูปที่ 2**.

BRAIN Lab
สำนักวิชาวิทยาศาสตร์และเทคโนโลยีสารสนเทศ (IST)
สถาบันวิทยสิริเมธี (VISTEC)
Phase1: การพัฒนาระบบอัจฉริยะ (Intelligent mode) ของ Exoskeleton
โมดูล 1: การพัฒนาส่วนปรับตัวได้ตามผลค่าความต่างของมุม
โมดูล 2: การพัฒนาระบบ CPG
โมดูล 3: การพัฒนาระบบสร้างรูปแบบการเคลื่อนที่

→

Gait Analysis Lab
ภาควิชาออร์โธปิดิกส์
โรงพยาบาลรามาธิบดี
Phase2: การเปรียบเทียบระบบอัจฉริยะ (Intelligent mode) กับ ระบบควบคุมพื้นฐาน (Default mode) ของชุดโครงกระดูกภายนอก ในกลุ่มอาสาสมัครสุขภาพดี
Phase 2.1: การทดสอบระบบ Intelligent mode เพื่อการช่วยเหลือ (Assistive purpose)

รูปที่ 2 รูปแบบการวิจัย

7.4 ขั้นตอนการเก็บข้อมูล

7.4.1 สำหรับ Phase1 การพัฒนาระบบอัจฉริยะของชุดโครงกระดูกภายนอกที่ สถาบัน VISTEC

7.4.2 สำหรับ Phase2 การศึกษาผลของระบบพัฒนาอัจริยะแบบช่วยเหลือ เปรียบเทียบกับระบบพื้นฐานของชุดโครงกระดูกภายนอก ที่ห้องปฏิบัติการวิเคราะห์การเดิน โรงพยาบาลรามาธิบดีอาสาสมัครที่ยินยอมเข้าร่วมการวิจัยจะได้รับการอธิบายวัตถุประสงค์ของโครงการวิจัย, ข้อมูลเกี่ยวกับ ขั้นตอนการวิจัย, ข้อห้ามข้อควรระวังเกี่ยวกับความเสี่ยงที่อาจเกิดขึ้น, และค่าชดเชยที่อาสาสมัครจะได้รับ จากนั้นอาสาสมัครจะต้องเซ็นเอกสารยินยอมเข้าร่วมงานวิจัย และกรอกข้อมูลส่วนตัวพื้นฐาน เช่น อายุ น้ำหนัก ส่วนสูง และประวัติการเจ็บป่วยของร่างกาย เป็นต้น

โดยอาสาสมัครจะทดสอบการเดินขณะยังไม่สวมใส่อุปกรณ์โครงกระดูกภายนอกเพื่อเก็บข้อมูลการเดินเบื้องต้น จากนั้นผู้วิจัยจะเปิดซองจดหมายที่ได้รับการสุ่มลำดับการเดิน และอาสาสมัครจะต้องเดินตามลำดับที่สุ่มได้อย่างใดอย่างหนึ่ง

- **รูปแบบการสุ่มที่1** อาสาสมัครทดสอบเดินด้วยชุดโครงกระดูกภายนอกในโหมดควบคุมแบบพื้นฐาน แล้วจึงพัก 10 นาที หลังจากนั้นอาสาสมัครทดสอบเดินด้วยชุดโครงกระดูกภายนอกในโหมดควบคุมอัจฉริยะแบบช่วยเหลือ (assist mode)
- **รูปแบบการสุ่มที่2** อาสาสมัครทดสอบเดินด้วยชุดโครงกระดูกภายนอกในโหมดควบคุมอัจฉริยะแบบช่วยเหลือ (assist mode)แล้วจึงพัก 10 นาที หลังจากนั้น อาสาสมัครทดสอบเดินด้วยโครงกระดูกภายนอกในโหมดควบคุมแบบพื้นฐาน

หลังจากทราบลำดับการสุ่มแล้ว อาสาสมัครจะต้องสวมใส่ชุดโครงกระดูกภายนอก ที่มีน้ำหนัก 11 กิโลกรัม สูง 118 เซนติเมตร โดยผู้วิจัยจะคอยดูแลขณะอาสาสมัครสวมใส่ รวมถึงให้ทดลองเดินจนกว่าอาสาสมัครจะสามารถเดินเองได้อย่างมั่นใจ แล้วจึงทำการทดสอบการเดินด้วยเครื่องวิเคราะห์การเดิน

7.5 ขั้นตอนการติดอุปกรณ์และการตรวจวิเคราะห์การเดิน

ทางทีมผู้วิจัยจะนำชุดมาให้อาสาสมัครเปลี่ยน โดยเป็นเสื้อและกางเกงขาสั้น เพื่อสะดวกต่อการติดอุปกรณ์ตรวจวิเคราะห์การเดิน และ เครื่องวัดสัญญาณกล้ามเนื้อที่บริเวณผิวหนัง โดยผู้วิจัยจะทำการติดอุปกรณ์สะท้อนแสง (reflective marker) ที่ตัวของอาสาสมัคร จำนวน 29 จุด ตามวิธีการของ Helen Heys model ได้แก่ บริเวณด้านหน้าศีรษะ (Frontal Head), ด้านบนศีรษะ (Top Head), ด้านหลังศีรษะ (Rear Head), หัวไหล่ขวา (Right Shoulder), กระดูกไหปลาร้าขวา (Off set), ข้อศอกขวา (Right Elbow), ข้อมือขวา (Right Wrist), หัวไหล่ซ้าย (Left Shoulder), ข้อศอกซ้าย (Left Elbow), ข้อมือซ้าย (Left Wrist), ปุ่มกระดูกเชิงกรานด้านหน้าข้างขวา (Right Anterior Superior IliacSpine), ปุ่มกระดูกเชิงกรานด้านหน้าข้างซ้าย (Left Anterior Superior IliacSpine), กระดูกสันหลังระดับ S1 (S1 Spine), ต้นขาขวา (Right Thigh), ข้อเข่าขวาด้านนอก (Right Lateral Knee), ข้อเข่าขวาด้านใน (Right Medial Knee), หน้าแข้งขวา (Right Shank), ข้อเท้าขวาด้านนอก (Right Lateral Ankle), ส้นเท้าขวา (Right Heel), นิ้วหัวแม่เท้าขวา (Right Toe), ข้อเท้าขวาด้านใน (Right Medial Ankle), ต้นขาซ้าย (Left Thigh), ข้อเข่าซ้ายด้านนอก (Left Lateral Knee), ข้อเข่าซ้ายด้านใน (Left Medial Knee), หน้าแข้งซ้าย (Left Shank), ข้อเท้าซ้ายด้านนอก (Left Lateral Ankle), ส้นเท้าซ้าย (Left Heel), นิ้วหัวแม่เท้าซ้าย (Left Fore Foot), ข้อเท้าซ้ายด้านใน (Left Medial Ankle)

ติดอุปกรณ์วัดสัญญาณกล้ามเนื้อไฟฟ้า โดยใช้แผ่นแปะขั้วไฟฟ้า Bipolar Ag/AgCl (3M Red dot, size 35x40 cm) ติดที่บริเวณผิวหนังตามตำแหน่งของสะโพก กล้ามเนื้อ , ก้น, หน้าขา, หลังขา, หน้าแข้ง และน่องด้านหลัง บนผิวหนังตามตำแหน่งกล้ามเนื้อทั้งสองข้างได้แก่ บริเวณสะโพก ได้แก่ กล้ามเนื้อกลูเตียส (Gluteal muscles), บริเวณหน้าขา ได้แก่ กล้ามเนื้อเรกตัส ฟีเมอริส (Rectus femoris), บริเวณหลังขา ได้แก่ กล้ามเนื้อไบเซบฟีเมอริส (Biceps Femoris), บริเวณหน้าแข้งทางด้านหน้า ได้แก่ กล้ามเนื้อทิเบียลิส แอนเทอเรีย (Tibialis anterior) และน่องทางด้านหลัง ได้แก่ กล้ามเนื้อแกสตอกนีเมียส (Medial Gastrocmuis) หลังจากนั้นติดอุปกรณ์วัดสัญญาณกล้ามเนื้อ Myon 320 wireless EMG(Myon AG, Schwarzenberg Switzerland) ตามบริเวณที่แปะแผ่นวัดสัญญาณกล้ามเนื้อ โดยใช้สายยางรัดสีดำเป็นตัวยึดอุปกรณ์

อาสาสมัครจะต้องเดินเป็นเส้นตรงไปและกลับ ด้วยระยะทางประมาณ 8 เมตร เป็นเวลาประมาณ 5 นาที การทดสอบแรกอาสาสมัครจะทำการเดินแบบยังไม่สวมใส่ชุดโครงกระดูกภายนอกเพื่อเก็บข้อมูลเบื้องต้นของแต่ละบุคคลก่อน จากนั้นจึงให้เดินโดยสวมใส่ชุดโครงกระดูกภายนอก ดัง**รูปที่ 3**โดยอาสาสมัครจะได้รับคำแนะนำในการใช้อุปกรณ์ และทดลองเดินจนกว่าจะคุ้นชินกับอุปกรณ์ก่อนทำการทดสอบจริง และจะมีผู้วิจัย 2 คนคอยประกบดูแลความปลอดภัยตลอดเวลา

ขณะทดสอบข้อมูลวิเคราะห์การเดิน การทำงานของกล้ามเนื้อ และการวัดค่าการใช้ออกซิเจน จะถูกบันทึกโดยอุปกรณ์การทดสอบการเดินด้วยเครื่องวิเคราะห์การเดินด้วยเครื่อง3D motion capture and Cortex 6.2 software ดัง**รูปที่ 4**,ทดสอบค่าการทำงานของกล้ามเนื้อขณะเดินด้วยเครื่องวัดสัญญาณกล้ามเนื้อ(Electromyography) ด้วยเครื่อง Myon 320 wireless EMG ดัง**รูปที่ 5**,และวัดค่าการใช้ออกซิเจนด้วยเครื่องวัดการใช้ออกซิเจน(Oxygen Consumption) ดัง**รูปที่ 6** ตามลำดับ

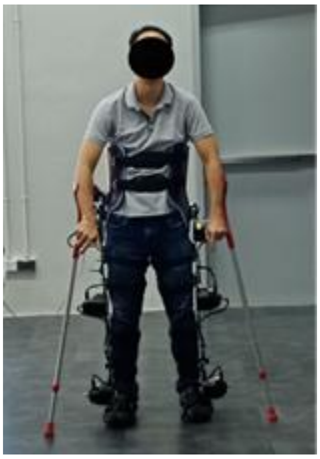

รูปที่ 3 ตัวอย่างการสวมใส่ชุดโครงกระดูกภายนอก

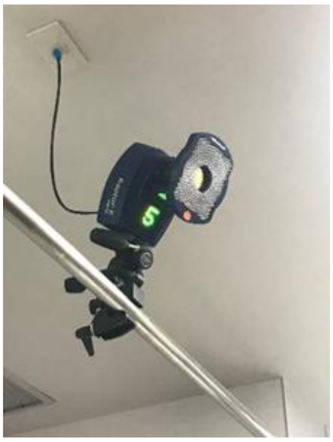

รูปที่ 4 เครื่องวิเคราะห์การเดิน 3D motion capture

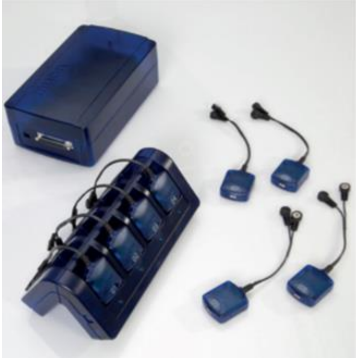

รูปที่ 5 เครื่องตรวจวัดสัญญาณกล้ามเนื้อ

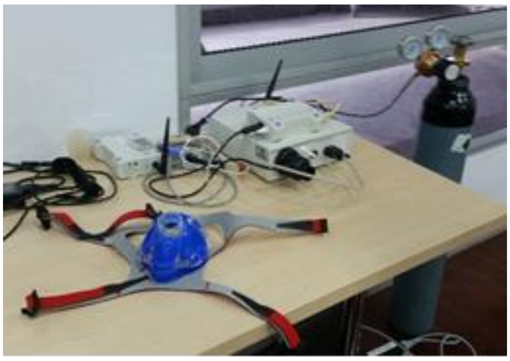

รูปที่ 6 เครื่องวัดการใช้ออกซิเจน

โดยหลังจากเสร็จการทดสอบแล้ว ผู้วิจัยจะทำการประเมินประสิทธิภาพของโครงกระดูกภายนอก และความสะดวกสบายในการใช้โครงกระดูกภายนอก จากแบบสอบถามที่เตรียมไว้ โดยระหว่างที่จบการทดสอบแต่ละเงื่อนไข อาสาสมัครจะนั่งพักเป็นเวลา 10 นาที หรือจนกว่าจะหายเหนื่อย ก่อนที่จะเริ่มการทดสอบเงื่อนไขต่อไป

ระยะเวลาในการศึกษา

ระยะเวลาในการศึกษาแบ่งออกตามส่วนของงานวิจัย โดยงานวิจัยถูกแบ่งออกเป็นสามส่วน ดังนี้

- งานวิจัย Phase 1: การพัฒนาระบบ Exoskeleton : มกราคม 2563–ธันวาคม 2563
- งานวิจัย Phase 2: การทดสอบ Assistive mode ในกลุ่มอาสาสมัครสุขภาพดี: มกราคม 2564 - ธันวาคม 2564

Time frame Phase 1

งานวิจัย Phase 1	หัวข้อ	พ.ศ. 2563											
		เดือน											
		1	2	3	4	5	6	7	8	9	10	11	12
การเตรียมข้อมูล	พัฒนา Proposal	x	x										
	การประชุมทีมวิจัย VISTEC & RAMA	x	x	x	x	x	x	x	x	x	x	x	x
ส่วนที่1	เตรียมอุปกรณ์	x	x	x	x	x	x						
	การเริ่มพัฒนาระบบ			x	x	x	x	x	x	x	x	x	x
	วิเคราะห์ผล											x	x
	เขียน manuscript										x	x	x

Time frame Phase 2

งานวิจัย Phase 2	หัวข้อ	พ.ศ. 2564											
		เดือน											
		1	2	3	4	5	6	7	8	9	10	11	12
การเตรียมข้อมูล	พัฒนา Proposal	x											
	การขอจริยธรรมในคน	x	x										
ส่วนที่1	เตรียมอุปกรณ์		x	x									
	เก็บข้อมูล				x	x	x	x	x	x	x	x	
	วิเคราะห์ผล											x	x
	เขียน manuscript											x	x

8. Protocol Flow Chart

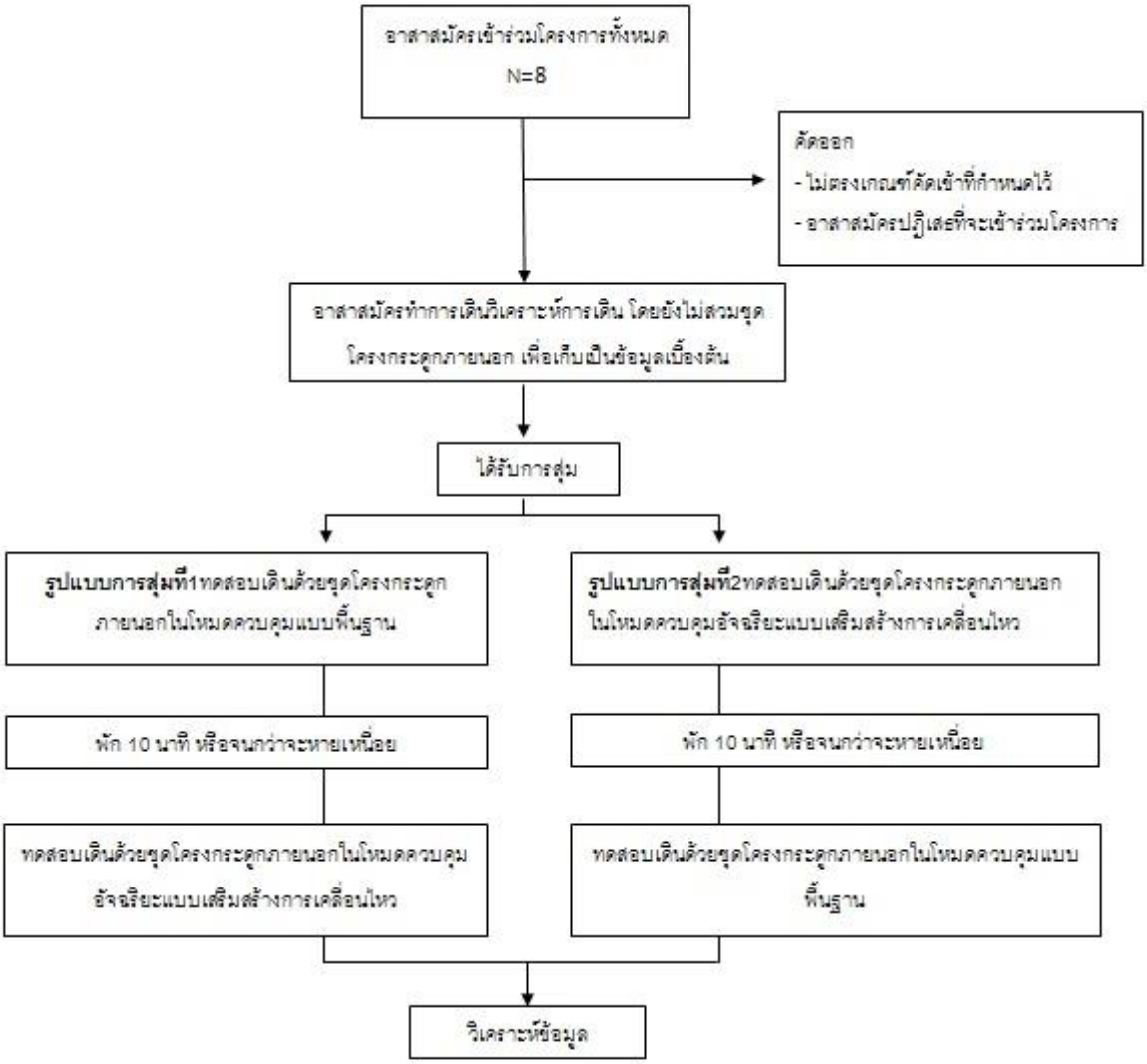

9. ระบุจำนวนอาสาสมัคร (Subject) ที่จะศึกษา พร้อมทั้งเกณฑ์คัดเข้าและเกณฑ์คัดออก

ขนาดกลุ่มประชากร(Sample Size)

ขนาดของกลุ่มประชากรคำนวณโดยอ้างอิงจากตัวอย่างงานวิจัย "Lower limb joint movement change in stroke patient with and without Knee Ankle Foot Orthosis (KAFO)." โดยใช้ค่า Mean และ SD ของขาข้างปกติ ในกลุ่มผู้ป่วยอัมพาตครึ่งซีก และเปรียบเทียบในขณะที่สวมใส่และไม่สวมใส่อุปกรณ์knee ankle foot orthotic *(*KAFO*)* **ดังตารางที่ 1**

ตารางที่ 1. ตารางการคำนวณขนาดกลุ่มประชากรตัวอย่าง

Alpha	Power	Meansd0	Del	Meansd1	SD diff	N per group	N total
0.05	0.8	5.1(7.4)	-2.9	2.2(6.1)	1.3	4	8
0.05	0.8	40.0(9.1)	-1.7	38.3(6.4)	2.7	22	44
**0.05**	**0.8**	**-0.8(7.6)**	**1**	**0.2(7.2)**	**0.4**	**4**	**8**
0.05	0.8	18.3 (7.1)	1.1	19.4(5.4)	1.7	21	42

Meansd0 = Mean and SD of the unaffected leg in stroke patient without KAFO

Meansd1 = Mean and SD of the unaffected leg in stroke patient with wearing KAFO

SD diff = The difference of standard deviation

Del = Mean difference between stroke patient with and without KAFO

ดังนั้น จะได้จำนวนอาสาสมัครที่มีความเป็นไปได้ คือทั้งหมด 8 คน แบ่งเป็นกลุ่มละ 4 คน

เกณฑ์การคัดเลือกอาสาสมัคร

งานวิจัยส่วนที่ 2 (อาสาสมัครสุขภาพดี)

<u>เกณฑ์การคัดเลือกอาสาสมัครเข้าโครงการวิจัย</u>

- เพศชายและหญิง อายุระหว่าง 18-60 ปีบริบูรณ์
- ดัชนีมวลกาย (Body mass index) อยู่ระหว่าง 18.5-24.9 กิโลกรัมต่อตารางเมตร
- ไม่มีประวัติการได้รับการผ่าตัดบริเวณหลัง ข้อสะโพก ข้อเข่า และข้อเท้า
- ไม่มีประวัติการบาดเจ็บบริเวณหลัง ข้อสะโพก ข้อเข่า และข้อเท้า ที่จะส่งผลต่อการศึกษา ในระยะเวลา 6 เดือนที่ผ่านมา
- ไม่มีประวัติเกี่ยวกับโรคทางประสาทกล้ามเนื้อที่จะส่งผลต่อการศึกษา เช่น *multiple sclerosis,* myasthenia gravis
- ยินดีเข้าร่วมโครงการวิจัยโดยการลงนาม

<u>เกณฑ์การคัดเลือกอาสาสมัครออกจากโครงการวิจัย</u>

- มีอาการบาดเจ็บบริเวณหลังส่วนล่างแบบเฉียบพลัน
- มีพยาธิสภาพที่สมอง
- มีปัญหาทางด้านการควบคุมการทรงตัว
- อาสาสมัครปฏิเสธ หรือขอถอนตัวออกจากการเข้าร่วมโครงการวิจัย

10. ระยะเวลาในการศึกษา(ต้องเริ่มหลังจากได้รับการอนุมัติจากคณะกรรมการจริยธรรมการวิจัยในคนแล้ว)

หลังจากได้รับอนุมัติจริยธรรมการวิจัยในคนเป็นระยะเวลา 2 ปี

11. ความเสี่ยงหรือความไม่สบายที่คาดว่าจะเกิดขึ้นกับอาสาสมัคร

ความเสี่ยงของอุปกรณ์ที่สัมผัสกับผิวหนังของอาสาสมัคร โดยปกติแล้วอุปกรณ์ที่ติดบนผิวหนังของอาสาสมัครเป็นเพียงการแปะลงบนผิวหนัง และจะไม่ก่อให้เกิดความรู้สึกเจ็บใดๆ แต่เนื่องด้วยอุปกรณ์สัมผัสกับผิวหนังของอาสาสมัครโดยตรง จึงอาจมีความเสี่ยงที่อาจจะเกิดการระคายเคืองหรืออาการแพ้บริเวณผิวหนัง โดยผู้วิจัยจะทำการสอบถามเบื้องต้นและตรวจสอบบริเวณที่อุปกรณ์สัมผัสกับผิวหนังของอาสาสมัครอยู่เป็นระยะ และหากมีอาการระคายเคืองหรืออาการแพ้เกิดขึ้น ผู้วิจัยจะทำการประเมินอาการ และปฐมพยาบาลเบื้องต้นทันที

ความเสี่ยงจากความล้าระหว่างการเดิน เนื่องจากการทดลองนี้ อาสาสมัครจะต้องทำการเดินติดต่อกันเป็นระยะเวลา 5 นาที จึงอาจเกิดอาการเหนื่อย เมื่อยล้ากล้ามเนื้อขณะเดิน โดยผู้วิจัยจะทำการสอบถามความรู้สึกและประเมินอาการเหนื่อยของอาสาสมัครทั้งก่อน และระหว่างการเดินเพื่อความปลอดภัยของอาสาสมัคร

ความเสี่ยงทางด้านอุบัติเหตุระหว่างการตรวจวิเคราะห์การเดิน เช่น หกล้ม เนื่องจากอาสาสมัครอาจไม่คุ้นชินกับการเดินโดยสวมใส่ชุดโครงกระดูกภายนอก โดยทางผู้วิจัยจะทำการอธิบายวิธีการใช้งาน และให้อาสาสมัครทดลองเดินกับโครงกระดูกภายนอกจนเกิดความคุ้นชิน และมั่นใจสามารถเดินเองได้ โดยจะมีผู้วิจัย 2 คนคอยตามระวังอยู่ด้านข้างตลอดขณะสวมใส่ชุดโครงกระดูกภายนอก เพื่อป้องกันไม่ให้เกิดความเสี่ยงในการหกล้มได้

12. ประโยชน์ที่คาดว่าจะได้รับ

Phase1

ระบบ Exoskeleton ที่ได้รับการพัฒนาจะสามารถตอบสนองคำสั่งจากทางผู้ใช้ได้อย่างมีประสิทธิภาพในทุกท่าทางการเดิน โดยระบบทำงานแบบปรับตัวได้อย่างเรียลไทม์รวมถึงการเรียนรู้อัตโนมัติเพื่อให้เหมาะสมกับผู้ใช้แต่ละคนโดยไม่ต้องทำการปรับค่าต่างๆ ด้วยมือหรือใช้การคำนวณที่ซับซ้อนแบบออฟไลน์

Phase2

การพัฒนาโหมดการทำงานแบบช่วยเหลือ (Assist) จะมีประโยชน์ต่องานเวชศาสตร์ฟื้นฟูอย่างมากในการพัฒนาอุปกรณ์เพื่อช่วยเหลือฟื้นฟูผู้ป่วยที่มีปัญหาทางด้านการเดินให้มีประสิทธิภาพมากขึ้น

13. ข้อพิจารณาด้านจริยธรรม

อาสาสมัครจะได้รับการอธิบายถึง แนวทางการดำเนินงานวิจัยทุกขั้นตอน ประโยชน์ และโทษที่อาจจะเกิดขึ้นต่อผู้ป่วยอย่างละเอียด หากอาสาสมัครไม่ประสงค์ที่จะเข้าร่วมในงานวิจัยหรือต้องการยุติการเข้าร่วมงานวิจัยหลังจากดำเนินงานไปแล้ว สามารถแจ้งปฏิเสธ/ยุติการเข้าร่วม ได้ทันทีโดยไม่จำเป็นต้องชี้แจงเหตุผล

14. ค่าชดเชยแก่อาสาสมัคร(ตามความจำเป็นและเหมาะสม) ในกรณีที่เกิดอันตรายหรือผลอันไม่พึงประสงค์ ต่ออาสาสมัคร อาสาสมัครจะได้รับการดูแลรักษาโดยไม่ต้องเสียค่าใช้จ่ายอย่างใดบ้าง

อาสาสมัครจะได้รับค่าตอบแทน และค่าเดินทางมาร่วมงานวิจัย คนละ 1,000 บาท ในกรณีเกิดเหตุการณ์อันไม่พึงประสงค์ใดๆ อาสาสมัครจะได้รับการพยาบาลเบื้องต้นจากแพทย์และนักกายภาพบำบัดประจำโครงการวิจัย และหากต้องเข้าพักรักษาตัวที่โรงพยาบาลสืบเนื่องจากอาการอันไม่พึงประสงค์ที่เกิดจากการทดลอง อาสาสมัครจะได้รับการรักษาโดยไม่ต้องเสียค่าใช้จ่ายใดๆ

15. ระบุแหล่งทุนสนับสนุน ในกรณีที่ได้รับทุนสนับสนุนจากภาคเอกชนให้แจกแจงรายละเอียดของงบประมาณ และใส่ชื่อผู้ประสานงานของผู้ให้ทุน พร้อมเบอร์โทรศัพท์ที่สามารถติดต่อได้

โครงการวิจัยนี้เป็นการร่วมมือกันระหว่างสองสถาบัน ได้แก่ ภาควิชาออร์โธปิดิกส์ คณะแพทยศาสตร์โรงพยาบาลรามาธิบดี และ สำนักวิชาวิทยาศาสตร์และเทคโนโลยีสารสนเทศ (IST) ของสถาบันวิทยสิริเมธี (VISTEC) โดยในส่วนของระบบควบคุมและชุดโครงกระดูกภายนอกทาง VISTEC เป็นผู้สนับสนุน(ชื่อผู้ประสานงานคือนายชายชาญ อรรควุฒิวาณิชย์ เบอร์โทรศัพท์ติดต่อ 081-436-8333) ในส่วนของค่าหัตถการตรวจวิเคราะห์การเดิน สำหรับทดลองชุดโครงกระดูกภายนอก, ค่าเดินทางของอาสาสมัคร, ค่าใช้จ่ายอื่นๆในโครงการวิจัย ทางสถาบัน VISTEC เป็นผู้สนับสนุน และอยู่ในระหว่างการดำเนินการส่งยื่นพิจารณาทุน ผ่านหน่วยวิจัยทางคลินิก (Clinical Research Center)โดยมีตารางค่าใช้จ่ายทุนวิจัยส่วนที่ 2 ดัง**ตารางที่ 2** ในส่วนของกรรมสิทธิ์ในการพัฒนา ทั้งสองสถาบันถือว่าเป็นเจ้าของร่วมกัน โดยทางทีมวิจัยจะประสานกับศูนย์พัฒนานวัตกรรมทางการแพทย์ (Mind center) เพื่อช่วยดูแลเรื่องผลประโยชน์ และลิขสิทธิ์ โดยหากมีสัดส่วนและจัดสรรผลประโยชน์ในเชิงพาณิชย์จะมีการตกลงกันในภายหลัง

ตารางที่ 2 ตารางค่าใช้จ่ายของงานวิจัยที่ได้รับการสนับสนุนจาก VISTEC ส่วนที่ 2 มีงบประมาณ 240,000 บาท โดยในตารางแจกแจงเป็นค่าใช้จ่ายรายหัว ของอาสาสมัครแต่ละราย

	Visit	Total
**1. Investigator**		
Enrollment // informed consent	1000	1000
Adverse event	1000	1000
**2. Study Physical therapy**		
Physical Therapy assessment & attach the device to the subject	1000	1000
**3. Local Lab**		
Motion analysis + Electromyography	12700	12700
Pedobarograph	2000	2000
Oxygen Consumption	1500	1500
Questionnaire	500	500
Volunteer expense	1000	1000
Hospital service fee (gait lab visit)	100	100
**4. Other procedure**		
Office supplies/Admin supplies – Printing (CRF), Stationary, Photocopy, Telephone	1150	1150
Accessible medical record	2000	2000
E-Submit		10,000
Total cost per subject		23,950
20% over head cost per subject		4,790
**Total cost + over head (per subject)**		28,740
Total cost x 8 subjects		191,600
20% over head cost x 8 subjects		38,320
**Total cost + overhead cost (8 subjects)**		**239,920**

16. เอกสารชี้แจงข้อมูลและคำแนะนำแก่อาสาสมัคร (Patient/Participant Information Sheet)

- ตามเอกสารแนบ -

17. หนังสือยินยอมโดยได้รับการบอกกล่าวและเต็มใจ(Informed Consent Form)

- ตามเอกสารแนบ –

18. หลักฐานหรือข้อมูลอ้างอิง

1. The report of the disability situation in Thailand [Internet]. Department of Promotion and Development of Quality of Life of People with Disabilities. 2017 [cited 29 Jan 2018]. Available from: www.dep.go.th.

2. Wang S, Wang L, Meijneke C, van Asseldonk E, Hoellinger T, Cheron G, et al. Design and control of the MINDWALKER exoskeleton. IEEE transactions on neural systems and rehabilitation engineering : a publication of the IEEE Engineering in Medicine and Biology Society. 2015;23(2):277-86.
3. Suzuki K, Mito G, Kawamoto H, Hasegawa Y, Sankai Y. Intention-based walking support for paraplegia patients with Robot Suit HAL. Advanced Robotics. 2007;21(12):1441-69.
4. Bach Baunsgaard C, Vig Nissen U, Katrin Brust A, Frotzler A, Ribeill C, Kalke YB, et al. Gait training after spinal cord injury: safety, feasibility and gait function following 8 weeks of training with the exoskeletons from Ekso Bionics. Spinal cord. 2018;56(2):106-16.
5. Lerner ZF, Damiano DL, Bulea TC. The Effects of Exoskeleton Assisted Knee Extension on Lower-Extremity Gait Kinematics, Kinetics, and Muscle Activity in Children with Cerebral Palsy. Scientific reports. 2017;7(1):13512.
6. Rocca A, Pignat JM, Berney L, Johr J, Van de Ville D, Daniel RT, et al. Sympathetic activity and early mobilization in patients in intensive and intermediate care with severe brain injuries: a preliminary prospective randomized study. BMC neurology. 2016;16:169.
7. Louie DR, Eng JJ. Powered robotic exoskeletons in post-stroke rehabilitation of gait: a scoping review. Journal of neuroengineering and rehabilitation. 2016;13(1):53.
8. Lerner ZF, Damiano DL, Park HS, Gravunder AJ, Bulea TC. A Robotic Exoskeleton for Treatment of Crouch Gait in Children With Cerebral Palsy: Design and Initial Application. IEEE transactions on neural systems and rehabilitation engineering : a publication of the IEEE Engineering in Medicine and Biology Society. 2017;25(6):650-9.
9. Bortole M, Venkatakrishnan A, Zhu F, Moreno JC, Francisco GE, Pons JL, et al. The H2 robotic exoskeleton for gait rehabilitation after stroke: early findings from a clinical study. Journal of neuroengineering and rehabilitation. 2015;12:54.
10. Mackiewicz-Milewska M, Jung S, Kroszczyński AC, Mackiewicz-Nartowicz H, Serafin Z, Cisowska-Adamiak M, et al. Deep venous thrombosis in patients with chronic spinal cord injury. The journal of spinal cord medicine. 2016;39(4):400-4.
11. Do JG, Kim DH, Sung DH. Incidence of deep vein thrombosis after spinal cord injury in Korean patients at acute rehabilitation unit. J Korean Med Sci. 2013;28(9):1382-7.
12. Leizorovicz AJPoH, Thrombosis. Long-term consequences of deep vein thrombosis. 1998;28(Suppl. 3):1-7.
13. Evans G, Dufresne CR, Manson PNJAiwctjfp, healing. Surgical correction of pressure ulcers in an urban center: is it efficacious? 1994;7(1):40-6.

19. ลายเซ็นหัวหน้าโครงการวิจัย

ลงชื่อ..หัวหน้าโครงการ

(นางสาวธันยพร ปะทะธง)

วันที่..

20. ประวัติโดยย่อของหัวหน้าโครงการ

- ตามเอกสารแนบ -

21. ใบรับรองการอบรม GCP, ใบรับรองการอบรม CITI Program, ใบรับรองการอบรม NIDA Clinical Trials Networkหรือใบรับรองที่เกี่ยวข้องกับการอบรมด้านจริยธรรมการวิจัยในคน(แนบอย่างใดอย่างหนึ่ง)

- ตามเอกสารแนบ -

22. Case record form (ถ้ามี)

- ตามเอกสารแนบ -

23. แบบสอบถาม (ถ้ามี)

- ตามเอกสารแนบ -

24. เอกสารอื่นๆ (ถ้ามี)

- ตามเอกสารแนบ –

Research project proposal form for consideration by the Human Research Ethics Committee Faculty of Medicine Ramathibodi Hospital Mahidol University

(full version)

1. The research project name

The Development of an Exoskeleton System for Assisting and Enhancing Patients' Mobility

2. Head of the research project

2.1 The head of the research project of the Department of Orthopedics, Faculty of Medicine Ramathibodi Hospital

Miss Tanyaporn Phatthong

Qualification: B.Sc. (Physical Therapy)

Workplace: Department of Orthopedics Dick, Faculty of Medicine, Ramathibodi Hospital, Mahidol University

Contact location: Department of Orthopedics Dick, Faculty of Medicine, Ramathibodi Hospital, Mahidol University

Telephone: 66+ 081-491-6382

2.2 The head of the joint research project of School of Science and Information Technology (IST) Vidyasirimedhi Institute (VISTEC)

Prof. Dr. Poramate Manoonpong

Qualification: Professor

Workplace: School of Information Science and Technology (IST) of Vidyasirimedhi Institute

Contact location: School of Information Science and Technology (IST) of Vidyasirimedhi Institute (VISTEC)

555 Moo 1, Pa Yup Nai Subdistrict, Wang Chan District, Rayong Province 21210

Telephone: 095-709-2635

3. Research assistants

3.1 Assoc.Prof.Dr. Patarawan Woratanarat, M.D., Ph.D.

Workplace: Department of Orthopedics Dick, Faculty of Medicine, Ramathibodi Hospital, Mahidol University Qualification

Contact location: Department of Orthopedics Dick, Faculty of Medicine, Ramathibodi Hospital, Mahidol University

Telephone: 02-201-1589

3.2 Assist.Prof.Dr.Chanika Angsanunsukh, M.D.

Qualification: Assistant Professor

Workplace: Department of Orthopedics Dick, Faculty of Medicine, Ramathibodi Hospital, Mahidol University

Contact location: Department of Orthopedics Dick, Faculty of Medicine, Ramathibodi Hospital, Mahidol University

Telephone: 02-201-1589

3.3 Asst. Prof. Dr. Noratep Kulachote, M.D.

Qualification: Assistant Professor

Workplace: Department of Orthopedics Dick, Faculty of Medicine, Ramathibodi Hospital, Mahidol University

Contact location: Department of Orthopedics Dick, Faculty of Medicine, Ramathibodi Hospital, Mahidol University

Telephone: 02-201-1589

3.4 Miss Krongkaew Kaewkasikam

Qualification: B.Sc. (Physical Therapy)

Workplace: Department of Orthopedics Dick, Faculty of Medicine, Ramathibodi Hospital, Mahidol University

Contact location: Department of Orthopedics Dick, Faculty of Medicine, Ramathibodi Hospital, Mahidol University

Telephone: 02-201-0412

3.5 Mr. Chaichan Akkawutthivanich

Qualification: M.Sc. in Biomedical Engineering.

Workplace: School of Information Science and Technology (IST) of Vidyasirimedhi Institute

Contact location: School of Information Science and Technology (IST) of Vidyasirimedhi Institute (VISTEC)

555 Moo 1, Pa Yup Nai Subdistrict, Wang Chan District, Rayong Province 21210

Telephone: 081-436-8333

4. The doctor/person who will care for the volunteer (People who consent to do research) and can contact at least 2 people for 24 hours

4.1 Miss Thanyaporn Phatthong

Workplace: Department of Orthopedics Dick, Faculty of Medicine, Ramathibodi Hospital, Mahidol University

Telephone: 081-491-6382

4.2 Assist.Prof.Dr.Chanika Angsanansuk

Workplace: Department of Orthopedics Dick, Faculty of Medicine, Ramathibodi Hospital, Mahidol University

Telephone: 02-201-1589

5. Background and Rationale

In our daily life, many activities have done for living particularly is walking. Walking is a basic form of movement locomotion and transportation. By 878 888 of Thai people with movement disability was reported in 2017[1]. These condition affects to physical and mental health problem. The limitation of movement brings those people at the increasing risk of secondary complications e.g. pressure ulcer, muscle atrophy, osteoporosis[2]. The resolution to improve physical and mental health of those people is to develop the effective device which help them to regain the mobility to stand and walk keenly.

Medical evolution has varied assistive devices combine with the conventional therapy for improve the ability of patient with gait problem. In the past decade, robot was developed for using in the health care system[3]. Robotic exoskeleton which one of the walking assistive device that used to regain the mobility such as standing up, sitting down, walking etc. Several studies found the lower exoskeleton training was generally safe and feasible for the patient with gait problem e.g. in case of spinal cord injury, stroke, cerebral palsy, etc[4-9]. Thus, exoskeleton is a useful device for the health care system.

The effective of exoskeleton training depends on several factors such as the smoothness of exoskeleton movement and coordination. Whereas, most of current exoskeleton controllers are predefined joint trajectories without real-time online adaptation. In the other words, the exoskeleton joint movement have been programed not to be adjusted the circumstantial movement for each user immediately. Therefore, it is difficult to develop the smoothly movement interaction of exoskeleton with these controllers. To achieve the smoothly movement in exoskeleton, the force interaction controllers is required[10]. Furthermore, it requires manual control-parameter adjustment or offline control-parameter optimization which will result a device achieved.

Base on the reviewed article[10], the exoskeleton controller such as the model-based control, hierarchy-based control, physical parameters-based control, and usage-based control system have not been fully completed in the multiple energy-efficient gaits for different environment condition. Additionally, these controllers were not suitable for develop the oscillatory movement (continuous movement). However, the new control system called biologically inspired central pattern generator (CPGs) is the control system that suits for oscillatory movement and it can generate the natural walk and robust rhythmic gait pattern. Several researches used the CPGs as a key ingredient to control the exoskeleton. The overall results were good. However, some information still lacks on using the CPGs with online adaptation and the study is just the simulation[11-13].

In this study, investigators set a goal to develop the lower limb exoskeleton based on CPGs with a novel real-time online adaptation though fast dynamic synchronization. This study used the exoskeleton EXO-H3 (Technaid, Spain). The purpose of this study is to show benefit over previous studies in term of close-loop feedback, robustness and fast real-time online adaptation for individuals. Moreover, the investigator will integrate the adaptive CPGs-base control with dynamic movement primitive and a muscle model to achieve adaptive and dynamic interaction between user and device and to be able to generate multiple gaits for different condition. These benefits will improve natural walking and will help patients regain their essential daily life motions.

Based on the reasons above, the objective of study is to develop the exoskeleton's assistive mode for use with healthy subjects to evaluate the effectiveness of the development program, allowing users to work in real-time and interact with the device to restore natural walking.

6. Research Objective

This study set a goal to develop biologically-inspired control mechanism with fast real-time online adaptation for an adaptive exoskeleton and see the effectiveness compare with the basic control by using the measurement of Gait Analysis Laboratory which consist motion analysis, electromyography, and oxygen consumption as the following;

6.1 Primary objective

6.1.1 To study the average peak kinematic values of hip, pelvic, knee and ankle in individuals with wearing lower exoskeleton compare between basic control mode/default mode and the intelligent mode of exoskeleton

6.2 Secondary objective

6.2.1 To study the peak kinetic values of hip, knee and ankle in individuals with wearing lower exoskeleton compare between default mode and the intelligent mode.

6.2.2 To study the average temporal-spatio (gait parameters) in individuals with wearing lower exoskeleton compare between default mode and the intelligent mode

6.2.3 To study muscle activity of lower muscle extremities in individuals with wearing lower exoskeleton compare between default mode and the intelligent mode

6.2.4 To measure the use of oxygen consumption in individuals with wearing lower exoskeleton compare between default mode and the intelligent mode

6.2.5 To measure the performance of exoskeleton control system_compare between default mode and the intelligent mode

6.2.6 To study the comfort of the user during wearing the lower exoskeleton compare between default mode and the intelligent mode.

7. Methodology

7.1 Study design

2x2 Cross-over design

7.2 Setting& Period of study

Gait Analysis Laboratory, Floor4, Orthopedic building, Department of Orthopedics, Faculty of Medicine, Ramathibodi Hospital from June 2018 to December 2018.

7.3 Process

This project is a joint project between the Department of Orthopedics, Faculty of Medicine, Ramathibodi Hospital, Mahidol University and the School of Information Science and Technology (IST) of the Vidyasirimedhi Institute (VISTEC) to study the effectiveness of intelligent mode of the exoskeleton. The process is divided into 2 phases as follows:

Phase 1: Development of the exoskeleton's intelligent mode,

This phase will be carried out by a research team from Vidyasirimedhi Institute of Science and Technology (VISTEC) and will involve the development of three modules, as shown in **Figure 1**.

Module 1: Develop error-based adaptation module

Module 2: Develop basic movement based on Central Pattern Generator (CPG)

Module 3: Develop pattern generation for each individual user

After developing the exoskeleton system in three steps, VISTEC will take the developed exoskeleton system to test in Phase 2.

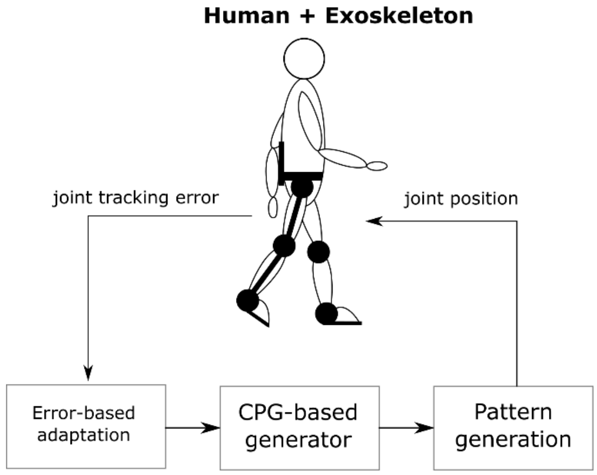

Figure 1 Exoskeleton system development by VISTEC

Phase 2: Testing the developed exoskeleton system with healthy subjects

This phase will be done at Gait Analysis Laboratory, Ramathibodi Hospital, Mahidol University (**Figure 2**). The experiment is as follows.

2.1 A study of the exoskeleton system with the **intelligent mode for assisting purpose** compared to the **default mode** of the exoskeleton in healthy subjects. Gait kinematics, muscle activity, oxygen consumption, exoskeleton performance, and user comfort while not wearing and wearing the lower-limb exoskeleton.

BRAIN Lab
School of Information Science and Technology (IST)
VISTEC
Phase1: Development of the intelligent mode for the exoskeleton
Module 1: Development of the error-based adaptation
Module 2: Development of the CPG-based generator
Module 3: Development of the pattern generator

Gait Analysis Lab
Department of Orthopedics
Ramathibodi Hospital
Phase2: Comparison of the intelligent mode with the default mode of the exoskeleton in healthy subjects
Testing the Intelligent mode for assistive purpose

Figure 2 Summary of research methodology

7.4 Data collection

The study is a collaboration effort between Gait Analysis Laboratory, Department of orthopedic, Ramathibodi Hospital and VISTEC.

7.4.1 Data for the development of the intelligent mode will be collected at the VISTEC Institute during Phase 1.

7.4.2 Data for Phases 2. will be collected at the Gait Analysis Laboratory at Ramathibodi Hospital. Phase 2 will examine the effects of the intelligent mode (assistive mode) compared to the default mode. The steps are as follows:

1) The researcher provides a detailed description of the study procedures and safety measure to all participants prior to participation. Each participant will then sign an informed consent form and fill out demographic data at the gait analysis laboratory. The demographic data collected from each participant will include sex, age, weight, height, BMI, occupation, religion, shoe size, underlying diseases, history of surgery, and history of lower limb injury.

2) Participants will undergo a motion analysis test by walking without the exoskeleton for 8 meters, back and forth, for approximately 15 rounds in the gait lab. The results will be used as the reference baseline to develop the intelligent mode (intervention) for each individual.

3) The researcher will open an envelope containing a random walking order (condition A/B) to determine whether the intervention will start with the intelligent mode or the default mode. Participants will then walk according to this random order.

Condition A is Individual walks with wearing the exoskeleton in **the intelligent mode.**

Condition B is Individual walks with wearing the exoskeleton in **the default mode**

In this study used the EXO-H3 exoskeleton (Technaid, Spain), weight of device is 11 kilograms, and it was covering since the waist to ankle of user. The maximum and minimum

user's weight are 100 and 45 kilograms, the maximum and minimum user's height are 195 and 140 centimeters, respectively.

4) Participants have to perform testing motion gait analysis, electromyography, and oxygen consumption for each condition. These measurements will be measured at the same time during individuals walking. The individuals have to walking in the straight line go and back, the distance around 8 meters, for 5 minutes. Then, rest for 10 minutes (To reduce bias of carry over effect). After that, start walking on the pedobarograph for 5 rounds.

5) After finishing testing, the investigator will assess the performance of the exoskeleton and the comfortable of the user by using the questionnaire.

7.5 Measurement

The investigator put the motion sensor on 29 bony prominence whole their body follow by the Halen heyes model which consist of frontal head, top head, rare head, right shoulder, clavicle, right elbow, right wrist, left shoulder, left elbow, left wrist, right anterior superior Iliac crest, left anterior superior Iliac crest, s1 spine, right thigh, right lateral knee, right medial knee, right shank, right lateral ankle, right medial ankle, right heel, right toe, left thigh, left lateral knee, left medial knee, left shank, left lateral ankle, left medial ankle, left heel, left toe.

The electrodes were placed parallel to the muscle fibers with an inter-electrode distance of 2 cm to avoid unstable recordings as recommended. The bipolar Ag/AgCl surface electrodes (3M Red dot, size 35 ×40 mm) were attached on 7 muscles at both side of each individuals including gluteus medius, gluteus maximus, rectus femoris, biceps femoris, tibialis anterior, and medial gastrocnemius. EMG signals were recorded by ProEMG software at 2000Hz using a Myon 320 wireless EMG (Myon AG, Schwarzenberg, Switzerland).

Participants will walk without wearing an exoskeleton to collect baseline data. They will then receive instructions to walk with an exoskeleton (**Figure 3**) in a straight line for 8

meters over 5 minutes. All participants will be instructed to practice walking with the exoskeleton until they become familiar with it. And researchers will be present to ensure safety. Gait parameters, muscle activity and oxygen consumption will be measured. Gait parameters will be recorded using 3D motion capture and Cortex 6.2 software, as shown in **Figure 4.** Muscle activity will be measured with electromyography using the Myon 320 wireless EMG (**Figure 5**). Oxygen consumption will measure with an oxycon mobile as (**Figure 6)**.

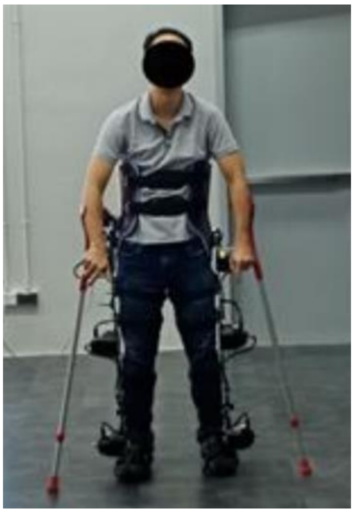

Figure 3. Examples of wearing an exoskeleton suit

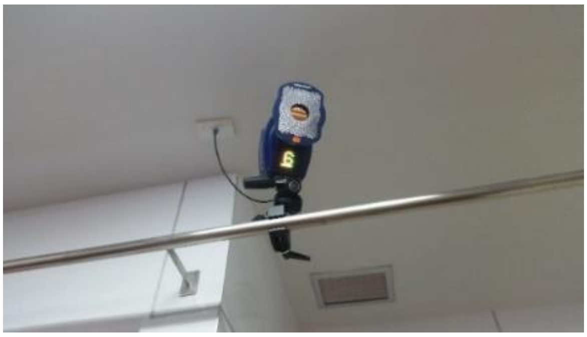

Figure 4. 3D motion capture

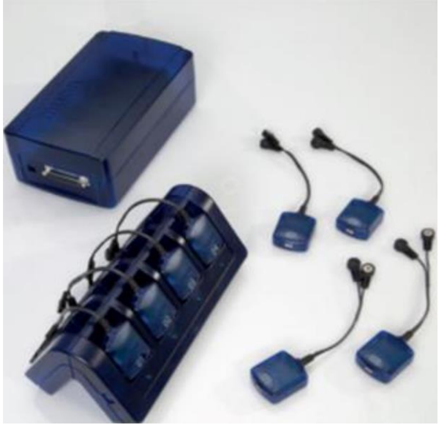

Figure 5. Myon 320 wireless EMG

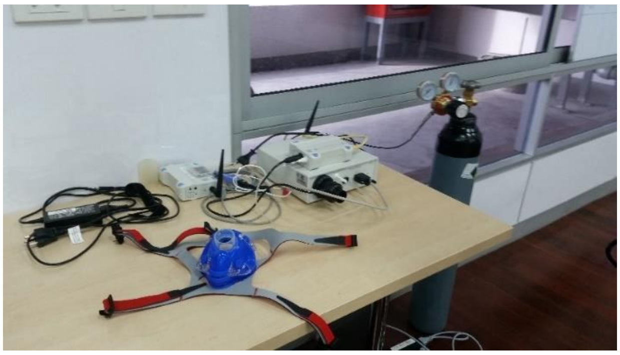

Figure 6. Oxycon mobile

After completing the test, participants will respond to questionnaires about the performance of the exoskeleton and their comfort while wearing it. Participants will be given a 10-min rest period in each condition.

Time frame

The study period is divided into three parts as follows.

- Phase 1: The development of exoskeleton system will be conducted from January to December 2020
- Phase 2: Testing of the effect of exoskeleton in intelligent mode (assistive mode) on healthy subjects will be conducted from January to December 2021.

Timeline for phase 1

phase 1	Details	Year 2020											
		month											
		1	2	3	4	5	6	7	8	9	10	11	12
	Proposal development	X	X										
	Ethical approval	X	X										
	Lab set up	X	X										
	Data collection			X	X	X	X	X	X	X	X	X	
	Data analysis										X	X	
	Writing manuscript											X	X

Timeline for phase 2

phase 2	Details	Year 2021											
		month											
		1	2	3	4	5	6	7	8	9	10	11	12
	Ethical approval	X	X										
	Data collection			X	X	X	X	X	X	X	X		
	Data analysis										X	X	
	Writing manuscript											X	X

8. Protocol Flow Chart

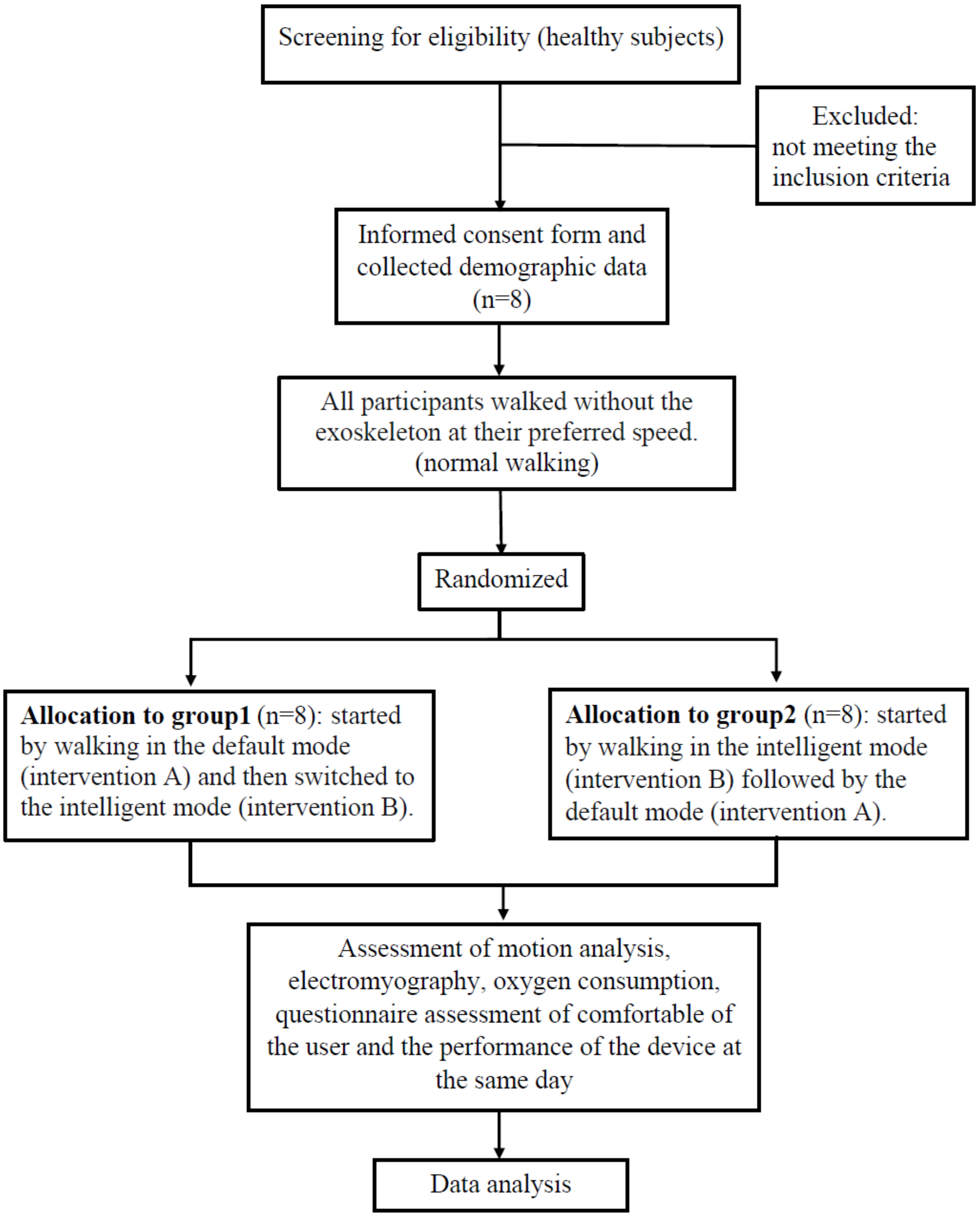

9. Sample size estimation

Sample size estimation is performed based on evidence for lower limb joint movement change in stroke patient with and without Knee Ankle Foot Orthosis (KAFO) is shown in **Table 1**.

The study will be measured in normal individuals. We used the mean and standard deviation from unaffected leg of the stroke patient compare between with and without knee ankle foot orthotic (KAFO). This study estimated the sample size based on these values and used type I error (alpha) of 0.05, power of 0.8.

Table 1. Sample size from various peak values of affected side of lower limb joint movement in stroke patient with and without KAFO orthosis

Alpha	Power	Meansd0	Del	Meansd1	SD diff	N per group	N total
0.05	0.8	5.1(7.4)	-2.9	2.2(6.1)	1.3	4	8
0.05	0.8	40.0(9.1)	-1.7	38.3(6.4)	2.7	22	44
**0.05**	**0.8**	**-0.8(7.6)**	**1**	**0.2(7.2)**	**0.4**	**4**	**8**
0.05	0.8	18.3 (7.1)	1.1	19.4(5.4)	1.7	21	42

Meansd0 = Mean and SD of the unaffected leg in stroke patient without KAFO

Meansd1 = Mean and SD of the unaffected leg in stroke patient with wearing KAFO

SD diff = The difference of standard deviation

Del = Mean difference between stroke patient with and without KAFO

Therefore, the total number of subjects for phase 2 is 8 people.

Study subjects for phase 2

Thai healthy adult who can walk independently without any assistive device which will be eligible to include in our study.

Inclusion criteria

- All Thai healthy whose age between 18-60 years old
- Who have normal body mass index (BMI)range between 18.5-24.9 kg/m^2
- Who did not have the history of the operation in area of back, hip, knee, and ankle

- Who did not have the history of previous injury or pain in area of hip, knee, and ankle which will affect to walking pattern within past 6 months.
- Who did not have the history of musculoskeletal or neuromuscular disease e.g. multiple sclerosis, myasthenia gravis.

Exclusion criteria

- Who have acute back pain
- Who have brain pathology
- Who have balance disorder
- Who reject or withdrawn of this study

10. Study period (must begin after approval from the Human Research Ethics Committee)

The study will be conducted for 2 years after receiving ethics approval.

11. Risk for the participants

The device will be attached to the individuals' skin, which might cause skin irritation. The investigator will regularly check the individuals' skin to prevent this symptom. Additionally, fatigue and muscle soreness may occur during walking because the study requires participants to perform multiple measurements and walk for 5 minutes. However, the research team is always concerned about any adverse events and will remain alert to prevent any such occurrences. The investigator will stand by all times during testing to prevent the risk of falling

12. Benefits

Phase1

The developed exoskeleton system can efficiently respond to the user in all walking patterns. The system can adapt in real-time and automatically learn from each user without requiring manual adjustments or complex calculations.

Phase2

The development of an assistive working mode of exoskeleton will be useful to rehabilitation for patients with walking problems.

13. Research ethics consideration

This research will be measuring in humans, so ethical consideration is required. First step, the investigator (physical therapist) will provide an information sheet and explain all detailed description of this research including the objective, protocol, benefit and risk that can be happen to the individuals. Prior to participation, the individuals have to sign the inform consent at gait analysis laboratory, Department of Orthopedics, Ramathibodi Hospital. All information about individuals will be kept confidential and the disclosure of information to the relevant agencies only if necessary for academic reasons.

14. Compensation (as necessary and appropriate) in the event of harm or adverse results.

Participants will be compensated, and travel expenses to attend the study will be covered up to 1,000 baht per person. Participants will receive initial care from doctors and physical therapists involved in the research project. If hospitalization is required due to an adverse event resulting from the experiment, the participant will receive treatment free of charge.

15. The source of funding. (In the case of private sector funding, please provide details of the budget.And include the name of the coordinator of the funder with a contact phone number.)

This research project is a collaboration between the Department of Orthopedics, Faculty of Medicine, Ramathibodi Hospital, and the School of Information Science and Technology (IST) at the Vidyasirimedhi Institute of Science and Technology (VISTEC). VISTEC supports the exoskeleton development (coordinator: Mr. Chaichan Arkawattiwanich, contact phone number: 081-436-8333). The costs of examinations, gait analysis for exoskeleton testing, volunteer travel expenses, and other research-related expenses are supported by VISTEC. Additionally, a funding request is being submitted through the Clinical Research Center.

The research funding cost table is shown in **Table 2**. Both institutions are considered joint owners of the development. The research team will coordinate with the Medical Innovation

Development Center (Mind Center) to manage benefits and copyrights. Any proportion and allocation of commercial benefits will be agreed upon at a later stage.

Table 2. Budget

**Details**	**Visit** (Thai bath)	**Total** (Thai bath)
**1. Investigator**	1,000	1,000
Enrollment // informed consent	1,000	1,000
Adverse event		
**2. Study Physical therapy**		
Physical Therapy assessment & attach the device to the subject	1,000	1,000
**3. Local Lab**		
Motion analysis + Electromyography	12,700	12,700
Pedobarograph	2,000	2,000
Oxygen Consumption	1,500	1,500
Questionnaire	500	500
Volunteer expense	1,000	1,000
Hospital service fee (gait lab visit)	100	100
**4. Other procedure**		
Office supplies/Admin supplies – Printing (CRF), Stationary, Photocopy, Telephone	1,150	1,150
Accessible medical record	2,000	2,000
E-Submit		10,000
Total cost per subject		23,950
20% overhead cost per subject		4,790
**Total cost + over head (per subject)**		**28,740**
Total cost x 8 subjects		191,600
20% overhead cost x 8 subjects		38,320
**Total cost + overhead cost (8 subjects)**		**239,920**

Therefore, the budget of this study is **239,920** Thai baths. The investigator plan to ask the grants from faculty of Medicine, Ramathibodi Hospital.

16. Patient/Participant Information Sheet

- As attached –

17. Informed Consent Form

- As attached –

18. References

1. The report of the disability situation in Thailand [Internet]. Department of Promotion and Development of Quality of Life of People with Disabilities. 2017 [cited 29 Jan 2018]. Available from: www.dep.go.th.
2. Wang S, Wang L, Meijneke C, van Asseldonk E, Hoellinger T, Cheron G, et al. Design and control of the MINDWALKER exoskeleton. IEEE transactions on neural systems and rehabilitation engineering : a publication of the IEEE Engineering in Medicine and Biology Society. 2015;23(2):277-86.
3. Suzuki K, Mito G, Kawamoto H, Hasegawa Y, Sankai Y. Intention-based walking support for paraplegia patients with Robot Suit HAL. Advanced Robotics. 2007;21(12):1441-69.
4. Bach Baunsgaard C, Vig Nissen U, Katrin Brust A, Frotzler A, Ribeill C, Kalke YB, et al. Gait training after spinal cord injury: safety, feasibility and gait function following 8 weeks of training with the exoskeletons from Ekso Bionics. Spinal cord. 2018;56(2):106-16.
5. Lerner ZF, Damiano DL, Bulea TC. The Effects of Exoskeleton Assisted Knee Extension on Lower-Extremity Gait Kinematics, Kinetics, and Muscle Activity in Children with Cerebral Palsy. Scientific reports. 2017;7(1):13512.

6. Rocca A, Pignat JM, Berney L, Johr J, Van de Ville D, Daniel RT, et al. Sympathetic activity and early mobilization in patients in intensive and intermediate care with severe brain injuries: a preliminary prospective randomized study. BMC neurology. 2016;16:169.

7. Louie DR, Eng JJ. Powered robotic exoskeletons in post-stroke rehabilitation of gait: a scoping review. Journal of neuroengineering and rehabilitation. 2016;13(1):53.

8. Lerner ZF, Damiano DL, Park HS, Gravunder AJ, Bulea TC. A Robotic Exoskeleton for Treatment of Crouch Gait in Children With Cerebral Palsy: Design and Initial Application. IEEE transactions on neural systems and rehabilitation engineering : a publication of the IEEE Engineering in Medicine and Biology Society. 2017;25(6):650-9.

9. Bortole M, Venkatakrishnan A, Zhu F, Moreno JC, Francisco GE, Pons JL, et al. The H2 robotic exoskeleton for gait rehabilitation after stroke: early findings from a clinical study. Journal of neuroengineering and rehabilitation. 2015;12:54.

10. Mackiewicz-Milewska M, Jung S, Kroszczyński AC, Mackiewicz-Nartowicz H, Serafin Z, Cisowska-Adamiak M, et al. Deep venous thrombosis in patients with chronic spinal cord injury. The journal of spinal cord medicine. 2016;39(4):400-4.

11. Do JG, Kim DH, Sung DH. Incidence of deep vein thrombosis after spinal cord injury in Korean patients at acute rehabilitation unit. J Korean Med Sci. 2013;28(9):1382-7.

12. Leizorovicz AJPoH, Thrombosis. Long-term consequences of deep vein thrombosis. 1998;28(Suppl. 3):1-7.

13. Evans G, Dufresne CR, Manson PNJAiwctjfp, healing. Surgical correction of pressure ulcers in an urban center: is it efficacious? 1994;7(1):40-6.

19. Signature of the research project leader

Signed..The research project leader

(Ms. Thanyaporn Patatong)

Date..

20. Brief biography of the project leader

- As attached -

21. GCP training certificate, CITI Program training certificate, NIDA Clinical Trials Network training certificate or certificate related to human research ethics training (attach one of them)

- As attached -

22. Case record form (if any)

- As attached -

23. Questionnaire (if any)

- As attached -

24. Other documents (if any)

- As attached -

(Patient/Participant Information Sheet)

**PROJECT**	The development of an intelligent exoskeleton system for assisting and enhancing patient's mobility
**INVESTIGATOR**	Miss Tanyaporn Patathong
**SETTING**	Gait Analysis Laboratory, Floor 4, Building 5, Department of Orthopedics, Faculty of medicine, Ramathibodi Hospital.

INVESTIGATORS CONTACT INFORMATION

Chanika Angsanuntsukh, M.D.	Tel: 02-201-1589
Patarawan Woratanarat, Associate Professor, PhD	Tel: 02-201-1589
Tanyaporn Patathong, PT	Tel: 02-201-0412

RESEARCH SPONSORED

Faculty of Medicine, Ramathibodi Hospital, Mahidol University.

OBJECTIVE

This study will develop an intelligent control system of exoskeleton to help patients with gait problem and measure the effectiveness of this intervention. By using the measurement of Gait Analysis Laboratory to measure the clinical outcome compare between the development mode and the standard basic control.

METHOD

After participation, the individuals have to sign inform consent at gait analysis laboratory. First step, the individuals have to perform walking without wearing exoskeleton for testing. Second step, individual have to perform walking with wearing the exoskeleton in 2 mode control of the exoskeleton

The resting time between each mode is 10 minutes. The exoskeleton call EXO-H3 (Technaid, Spain), weight of device is 11 kilograms, and it will be covering your body since the waist to ankle.

For each condition, the individual has to perform a walk of 8 meters go and back at gait lab room for testing motion gait analysis, electromyography, and oxygen consumption for 5 minutes.

After finishing the test, the investigator assessed the performance of the exoskeleton and the comfort of the user by using the questionnaire.

BENEFIT AND RISK THAT CAN BE HAPPEN TO PARTICIPANT

BENEFIT FOR THE PARTICIPANT

The participants will receive the analysis of walking data by the specialist doctor and physical therapist. The participant can be consulted about walking conditions from the research team at any time. Additionally, the participants' information will benefit the development of robotic research to help patients with walking problems.

RISK FOR THE PARTICIPANTS

The device will be attached to individuals 'skin that might cause skin irritation. The investigator will always check the individual's skin to prevent the symptoms. Additionally, the investigator will stay beside the individuals all the time during testing to prevent risk of falling. However, the researcher team is always concerned about any adverse events, and we will be stay alerted to prevent all advert event.

COMPENSATION

Participants will be compensated, and travel expenses to attend the study will be covered up to 1,000 baht per person. Participants will receive initial care from doctors and physical therapists involved in the research project. If hospitalization is required due to an adverse event resulting from the experiment, the participant will receive treatment free of charge

DATA CONFIDENTIALITY

The investigator will keep all data about the individual confidentially. The disclosure of information to the relevant agencies only if necessary for academic reasons.

If you have any concerns about your participation in this research, please contact us. Or you can contact the Ethical Research Officer, Faculty of Medicine, Ramathibodi Hospital

Tel: 02-201-1544

Informed Consent Form

PROJECT The development of an intelligent exoskeleton system for assisting and enhancing patient's mobility

INVESTIGATOR Miss Tanyaporn Patathong

*Subject name..

Ages ....Hospital number.......................................Subject number.....................

The researcher already explained to me the details of this project. I clearly understood all details of the research project, benefits, and risks that can happen to me. I accept all of the terms and conditions, and I agree to participate in this research. I know that if there are any problems, I can contact the researcher at any time. I have the right to withdraw from this project at any time without any affect to my treatment. Additionally, my personal information will be kept confidential. The disclosure of information to the relevant agencies only if necessary for academic reasons.

……………………………… (Subject's signature)

……………………………… (First Witness's name)

………………………………..(Second Witness's name)

Date of Signing………………………

Investigator explanation

The person indicated above fully understands all the details of this study including the objective (to development of the intelligent program to assisting the patient mobility), benefits and risks of this study that can happen during participation. Researchers 'contact numbers are provided. The investigator prepares the planning to prevent any adverse event(s) systematically.

…………………………………… (Investigator sign)

Date of Signing………………………

Criticize of this study

Advantage

- The design of this study is a 2x2 cross-over study, so the data will be compared within the individual and it is reducing the inter individual variability.

- Due to the study design, it used a small sample size, and it reduced the cost of the study.

- This study is the big project that we covered all importance outcome parameters that will be useful for the development of the exoskeleton such as kinetic values, kinematic value, temporal-spatio-parameters, lower muscle activities, oxygen consumption and heart rate, foot pressure distribution.

- The outcomes of this study were measured by using high quality standard measurements. Therefore, the data of this study will be precise and high quality.

Disadvantage

- This study design may cause the sequence effect and carry over effect. In this study the investigator plans to randomize the intervention sequence for each individual and give the appropriate resting time to reduce this bias.

- The analysis of this study design is more complex than in a parallel group design.

Case record form

Screening form for normal individuals

1. Age ☐☐

2. Weight ☐☐☐

3. Height ☐☐☐

4. Body mass index ☐☐.☐☐

5. Do you have the injury at back, hip, knee, or ankle within past six months

☐ 1. Yes ☐ 2. No ☐ 9. Missing

5.1 If yes, please specify the area of injury…………………………………………………………

5.2 If yes, please specify how long have you been injured…………………………………………

6. Have you ever had the operation at hip, knee, or ankle

☐ 1. Yes ☐ 2. No ☐ 9. Missing

6.1 If yes, please specify the area of the operation……………………………………………

7. Have you been diagnosed with musculoskeletal or neuromuscular diseases

☐ 1. Yes ☐ 2. No ☐ 9. Missing

7.1 If yes, please specify the disease…………………………………………………………….

8. Have you had balance disorder

☐ 1. Yes ☐ 2. No ☐ 9. Not know

Case record form

Case record form for normal individuals' demographics data

1. Subject ID ☐☐
2. Date of enrollment (DD/MM/25YY) ☐☐ / ☐☐ / ☐☐☐☐
3. Date of birth (DD/MM/25YY) ☐☐ / ☐☐ / ☐☐☐☐
4. Age ☐☐
5. Size of shoes ☐☐
6. Recipient sex

☐ 1. Male ☐ 2. Female ☐ 9. Missing

7. Education

☐ 1. Below elementary school ☐ 2. Elementary school ☐ 3. High school

☐ 4. Bachelor's degree ☐ 5. Master's degree or higher ☐ 9. Missing

8. Do you have any underlying disease

☐ 1. Yes ☐ 2. No ☐ 9. Missing

if yes, please specify the disease……………………………………………………………..

Clinical information

9. Weight (Kgs) ☐☐☐
10. Height (cms) ☐☐☐
11. Body mass index ☐☐.☐☐

Case record form

Clinical outcomes data

1.Subject ID ☐☐

2. Date of examination (DD/MM/25YY) ☐☐ / ☐☐ / ☐☐☐☐

Motion analysis data

Kinematic values

**Kinematics value**	**With exo+basic**		**With exo+……….**	
	**Rt**	**Lt**	**Rt**	**Lt**
**Stance phase**				
Hip extension peak				
Hip flexion peak				
Knee extension peak				
Knee flexion peak				
Ankle dorsiflexion peak				
Ankle dorsiflexion peak				
**Swing phase**				
Hip extension peak				
Hip flexion peak				
Knee extension peak				
Knee flexion peak				
Ankle dorsiflexion peak				
Ankle dorsiflexion peak				

Kinetic values

**Kinetic values**	**With exo+basic**		**With exo+………**	
	**Rt**	**Lt**	**Rt**	**Lt**
**Initial double contact**				
Hip moment				
Knee moment				
Ankle moment				
**Simple support**				
Hip moment				
Knee moment				
Ankle moment				
**Final double contact**				
Hip moment				
Knee moment				
Ankle moment				

Gait parameter

**Gait parameter**	**With exo+basic**		**With exo+………**	
	Rt	**Lt**	**Rt**	**Lt**
Velocity				
Stride length				
Cadence				
Width length				
Step length				
Swing time				
Stance time				

Electromyography

**Muscle activities values**	**With exo+basic**		**With exo+………**	
**Root mean square**	**Rt**	**Lt**	**Rt**	**Lt**
Gluteus medius				
Gluteus maximus				
Rectus femoris				
Biceps femoris				
Gastrocnemius (medial head)				
Tibialis anterior				
**Muscle activities values**	**With exo+basic**		**With exo+………**	
**Maximum voluntary contraction**	**Rt**	**Lt**	**Rt**	**Lt**
Gluteus medius				
Gluteus maximus				
Rectus femoris				
Biceps femoris				
Gastrocnemius (medial head)				
Tibialis anterior				

Oxygen consumption

**Heart rate(Beat/minute)**	**With exo+basic**	**With exo+………**
HR		
**Maximum oxygen consumption**	**With exo+basic**	**With exo+………**
VO2 MAX		

The comfortability

	With exo+basic	With exo+………
**Summary comfortable score**		

The performance of the device

	With exo+basic	With exo+………
**Summary performance score**		
