# Supplementary material for: Comprehensive multi-metric analysis of user experience and performance in adaptive and non-adaptive lower-limb exoskeletons
Source: PLoS One. 2025 Jan 9;20(1):e0313593. doi: 10.1371/journal.pone.0313593 (PMC11717227; doi:10.1371/journal.pone.0313593)
Supplement: S1 File — (DOCX) [file pone.0313593.s003.docx]

**S1 File. The detail of Exoskeleton and the development of intelligent exoskeleton.**

**Exoskeleton Hardware**

The developing exoskeleton hardware in this study is based on Exo-H3 (consecutive model from Exo-H2 developed by Technaid company, Spain) which is a powered, mobilized, and lower-limb type as a platform for research and clinical trials (**Fig 1**). This exoskeleton was designed to be used with a person who can still partially walk. The former model, Exo-H2, had been evaluated on participants with post-stroke hemiparesis [1]. It also had been tested on subjects with incomplete subacute spinal cord injury and its performance was safe and robust enough for gait rehabilitation [2]. The total weight of the exoskeleton is about 11 kg. It has six actuated joints (electrical motors) including hip, knee, and ankle on both legs. The hip joint can operate 30° backward and 105° forward. The knee joint is between 105° backward to 5° forward. The ankle joint is 30° in both directions. The maximum operating torque for each joint is around 40 Nm. This can be translated to assistive ability for a wearer with weight up to 100 kg. There are integrated joint and torque sensors (measured from the motor current) for each motor. Besides, an additional interaction torque sensor (measured from the direct interaction force between the user and exoskeleton) is installed for each joint. The exoskeleton supports the wearer’s trunk by its hip orthosis. Major legs structures as thigh and shank are restricted with adjustable coated aluminum alloy bars. The wearer needs to balance himself/herself in frontal plane by crutches.

By default, a microcontroller set developed by the factory is used to do a low-level control via Controller Area Network (CAN) protocol. A mobile computer, Intel® NUC7i7BNH, insides a backpack set developed by our team is used as a host for a mid-level control program which is an interface to translate high-level command into low-level motor control signals. Robot Operating System (ROS) protocol and Message Queuing Telemetry Transport (MQTT) are deployed between high- and mid-level to make our framework more universal, standard, and comfortable to connect to other equipment such as a treadmill.


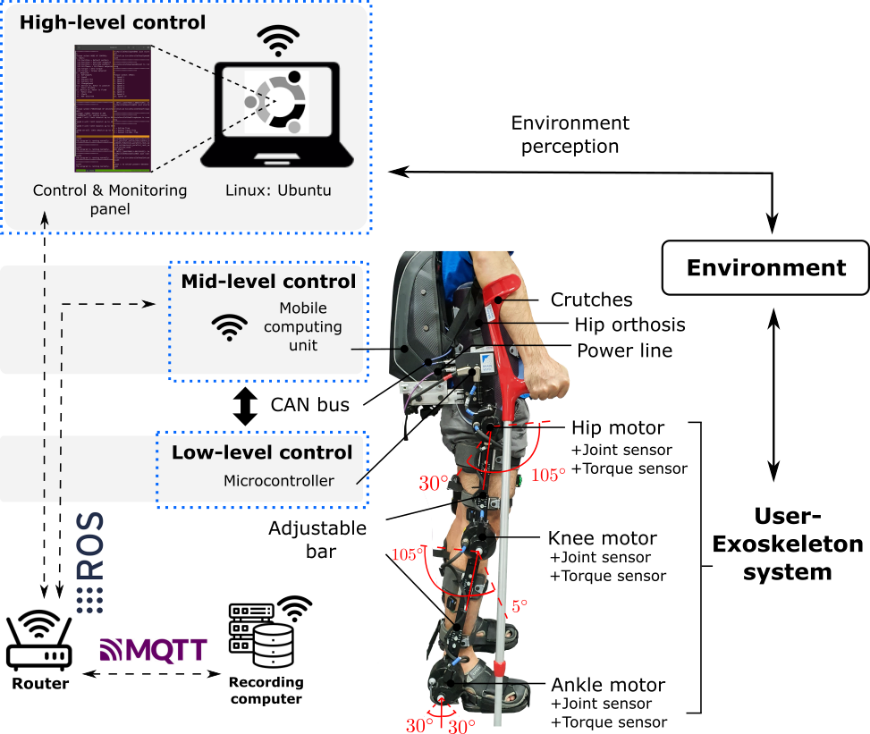


**Fig 1. Overview of our exoskeleton hardware and control system.** The exoskeleton hardware is a modified one based on Exo-H3 (Technaid, Spain) which is a sagittal-plane robot. There are three main control layers (High-, mid-, low-level). The system’s communication is based ROS and MQTT protocol.

**Control Algorithm**

We compare two control modes/algorithms in this study. The first one is a default control mode and the second one is an intelligent control mode [3]. Their control block diagrams are shown in **Fig 2**.


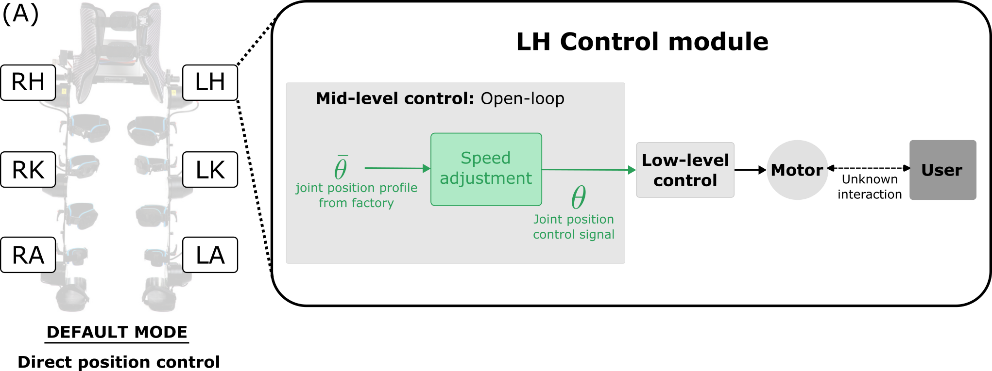


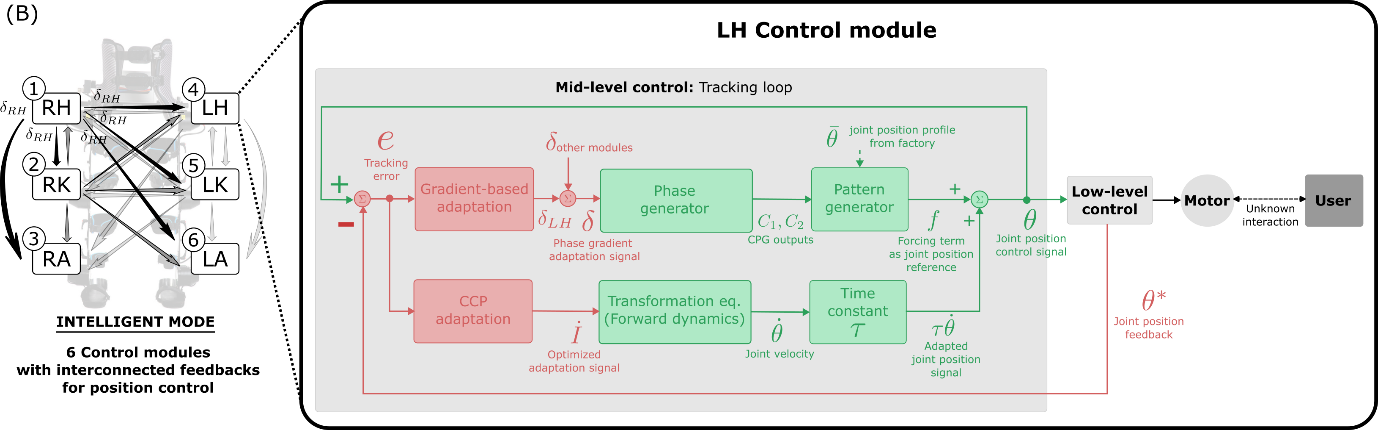


**Fig 2. (A) Block diagram of default control algorithm in this study.** The factory joint position profile is used to drive the exoskeleton with adjustable swing speed. **(B) Block diagram of intelligent control algorithm in this study.** It is Adaptive Modular Neural Control (AMNC) for online gait synchronization. RH/LH = Right/Left hip, RK/LK = Right/Left knee, and RA/LA = Right/left ankle.

For the default control mode (**Fig 2A**), joint position patterns ($\bar{\theta}$) of hip, knee, and ankle for both legs are obtained from the company. These joint patterns are average profiles collected from European subjects. When assist, our mid-level control algorithm samples the joint profile and directly sends each joint angle data ($\theta$) in an appropriate time to moderate a swing speed without any feedback information (open-loop control). The default control mechanism is designed for gait generation without adaptation during locomotion.

For the intelligent control mode[3], it performs as closed-loop control based on six neural control modules (modules 1-6, **Fig 2B.)** with interconnected feedback. Each module is designed to interact with other modules uniquely. The hip and knee modules share their feedback pathways ($\delta$) to all the other modules, while ankle modules only receive adaptation signals from the others. This coupling structure with bidirectional hip and knee feedback pathways is adopted to ensure a 90-degree phase difference between the left and right legs. The joint module (e.g., left hip module as an example in **Fig 2B.**) uses tracking loop concept where the algorithm adjusts its previous joint control signal to be suitable for a wearer in a current step. The module consists of six key components: phase generator, pattern generator, transformation equation (forward dynamics), time constant, gradient-based adaptation, and coupled cooperative primitives (CCP) adaptation. The phase generator creates rhythmic phase signals ($C_{1}$, $C_{2}$) based on a central pattern generator (CPG) concept. Those signals then activate the pattern generator module to produce a reference joint position angle at each time step ($f$) in this case for the left hip whose total profile/pattern is the joint position profile ($\bar{\theta}$) from the factory that has been previously learned and remembered by the module via weights of neural networks. The transformation eq. is then introduced with time constant block to create joint compliance on the system by making changes ($\tau\dot{\theta}$) on top of the reference joint position angle ($f$). An adaptation part comes from changing tracking error ($e= \theta-\theta^{*}$) into two forms to be used by the gradient-based adaptation and the CCP adaptation modules. The former uses the error to adjust phase of the control signal, while the latter regulates the joint compliant path. Note that the phase gradient adaptation signal ($\delta$) does not come only from the working module itself ($\delta_{LH}$), but also from the other modules ($\delta_{other modules}$) as mentioned earlier on the pathways. All in all, the intelligent control mechanism is designed for gait generation and adaptation in response to the tracking error during locomotion.

**Results and Discussion**

From the exoskeleton hardware point of view, there are four main variables that we mainly focus on in this study: swing speed (frequency), tracking error, joint trajectory, and interaction torque. When the intelligent algorithm works, we expect that the exoskeleton will smoothly follow the wearer’s intentional movement during the assistive period reducing spatial conflict between the exoskeleton and the wearer which in turn reflects as reduction in either tracking error or user-exoskeleton interaction torque as it has been shown in previous work [3]. The relationship between a pair of the tracking error or the interaction torque and swing speed will also be determined in this study.

**Speed and Frequency**

From the experiment, our eight subjects performed in different exoskeleton’s speeds and frequencies as shown in **Fig 3**. During the comfortable walk, most people tended to walk in a fast manner (L6; 0.333 Hz). A graph showing the comfortable swing frequency of each subject (**Fig 3B.**) looks the same when it is shifted to become fast values (up by ten percent). In contrast, the shape of the distribution when we categorize by speed looks a bit different when shifted due to different bin’s sizes. Higher speeds have wider size, so a frequency might fall into the same bin even if we have shifted by ten percent.


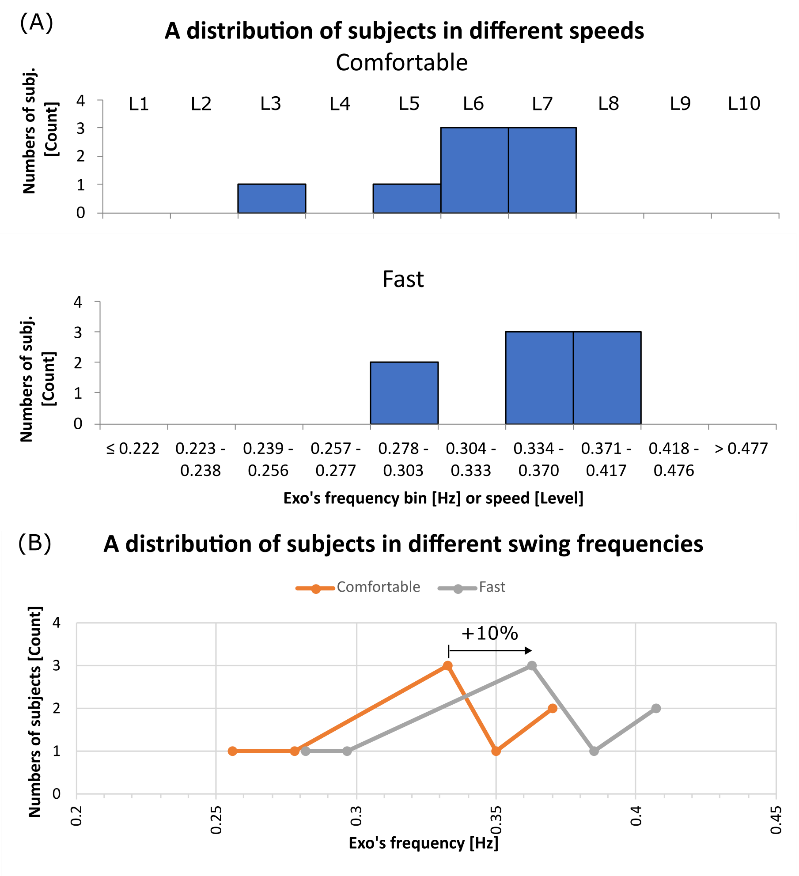


**Fig 3A. distribution of eight subjects over comfortable and fast (+10%) experiments is observed.** (A) A number of subjects categorized by speed level. (B) A number of subjects categorized by swing frequency.

## **Joint Tracking Error**

Regarding the tracking error, a root-mean-square (rms) error of hip, knee, and ankle of both default and intelligent modes are computed for each subject from:

$$e_{rms, j}= \sqrt{\frac{1}{N_{c}}\sum_{c=0}^{N_{c}} (\frac{1}{T_{c}}\sum_{t=0}^{T_{c}} \left( e_{j}^{c}\left[ t \right] \right)^{2})},$$

where

$j$ denotes the type of joint i.e., hip, knee, or ankle,

$c$ denotes the gait cycle index,

$t$ denotes the time step,

$e_{j}^{c}\left[ t \right]$ denotes the different between the joint position control signal and the joint position feedback at time step $t$, joint $j$, cycle $c$,

$T_{c}$ denotes the total number of time step in the $c^{th}$ gait cycle,

$N_{c}$ denotes the total number of gait cycle,

$e_{rms, j}$ denotes the rms error of either hip, knee, or ankle.

It is the calculation of each time step in one gait cycle, then calculate again across all gait cycles.

Then, a different score is defined as:

$$\Delta e_{rms,j}=e_{rms, j}^{I}-e_{rms, j}^{D},$$

where

$I$ denotes intelligent control algorithm,

$D$ denotes default control algorithm.

This $\Delta e_{rms,j}$ score represents the comparison between two control algorithms. Less values or more negative values means the intelligent control mode performs better than the default control mode due to low tracking error.

In **Fig. 4**, it shows the results from this defined different score for hip, knee, and ankle of both legs. All frequencies of all eight subjects in all slow, comfortable, and fast walking speed experiments are considered as data points. On average, the scores are below zero. This implies that our intelligent control algorithm outperforms the default control algorithm in term tracking error (error from intel. < error from default). The score also shows a downward trend when the swing frequency increases, so the intelligent control seems to work better on the fast-walking subjects. To have a control algorithm that focuses on dealing with different swing frequencies directly, we can refer to the work in [4].


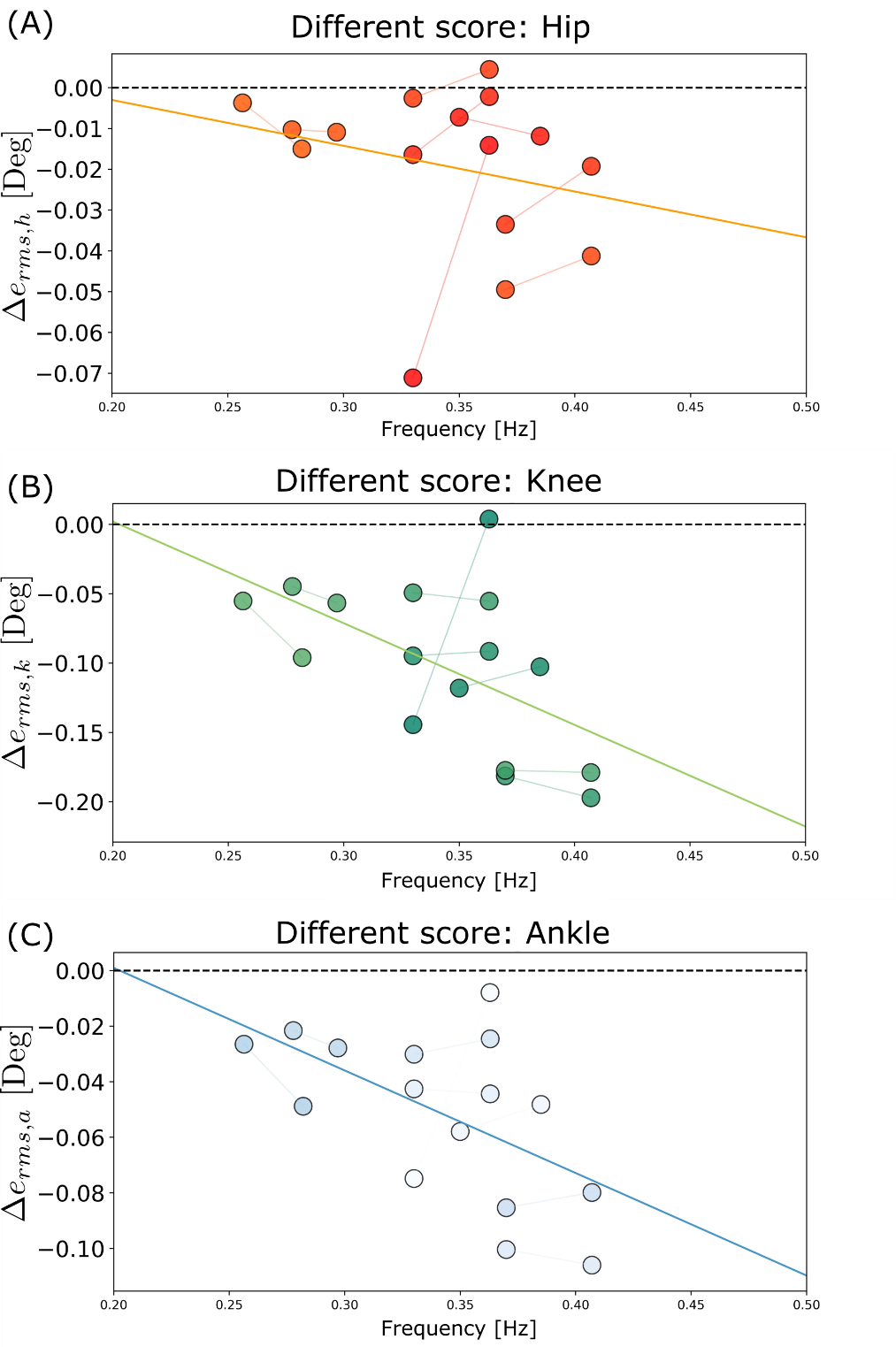


**Fig. 4 Different score or difference of rms of tracking error (**$\boldsymbol{\Delta}\boldsymbol{e}_{\boldsymbol{rms}}$**) between the intelligent mode and the default mode for hip, knee, and ankle.** Graph shows downward trend when the swing frequency increases. A group of dots connected by lines represents slow, comfortable, and fast values of a subject.

Individual rms tracking error of hip ($e_{rms, h}$), knee ($e_{rms, k}$), and ankle ($e_{rms, a}$) for eight subjects is shown in **Fig 5.** Due to the gait adaptation ability, the intelligent control mode mostly causes less rms tracking error compared to the default mode in all cases (comfortable or fast walking).


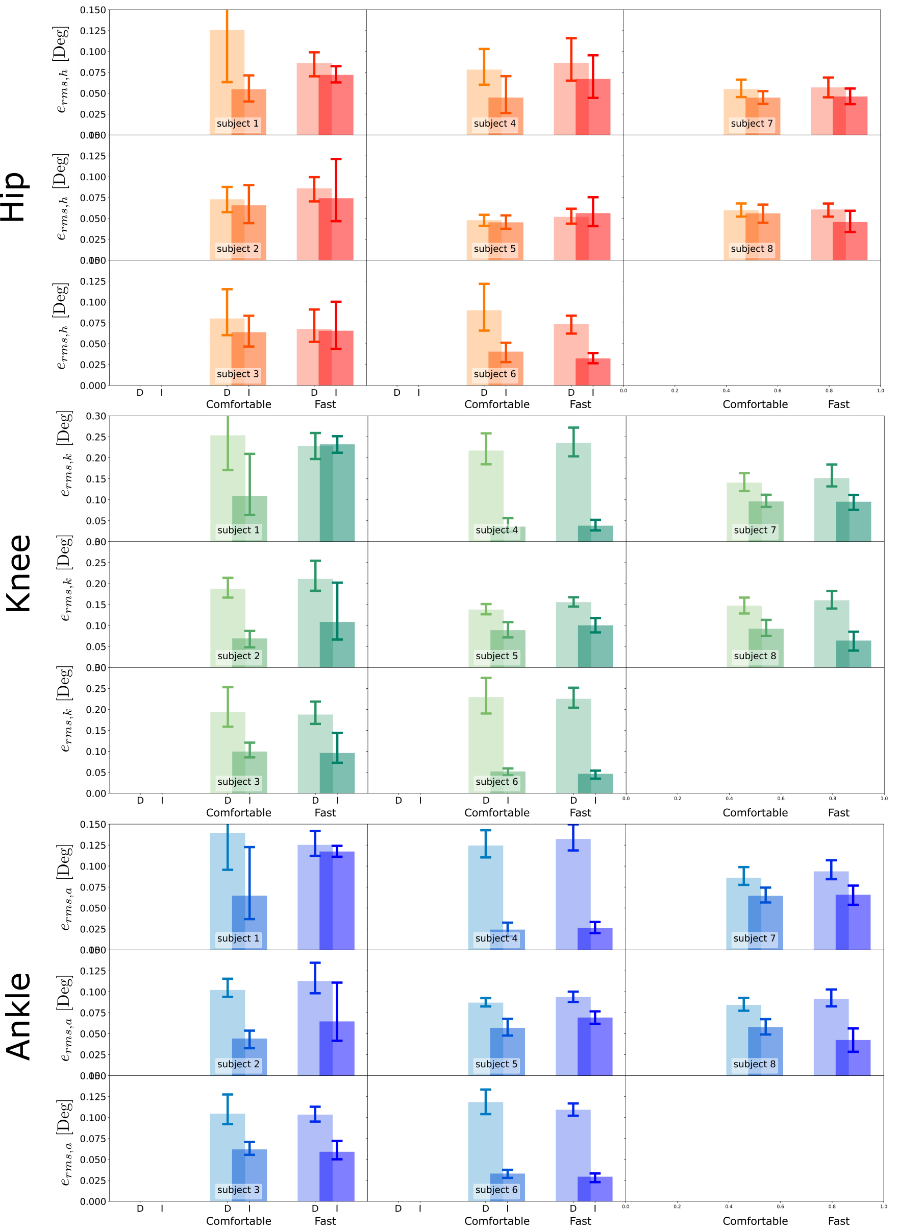


**Fig. 5 Individual rms tracking error of hip, knee, and ankle for eight subjects.** The error bar represents 80% confidence interval.

**Joint Trajectory**

Average joint trajectories of hip, knee, and ankle in one gait cycle of default and intelligent control modes during comfortable walking are compared in **Fig. 6** as an example. In general, the intelligent control mode still produces nearly the same joint trajectories (or patterns) for hip, knee and ankle except that their maximum amplitudes are lower. When average value of the difference score of hip ($\bar{\Delta e_{rms, h}}$) is used as a cutting point, two groups are classified between “good subject” and “bad subject” where the former has value below the average value and the latter has value above the average value. The good-subject group seems to have a little lower maximum amplitude than the bad-subject group in all hip, knee, and ankle trajectories in the intelligent control mode.


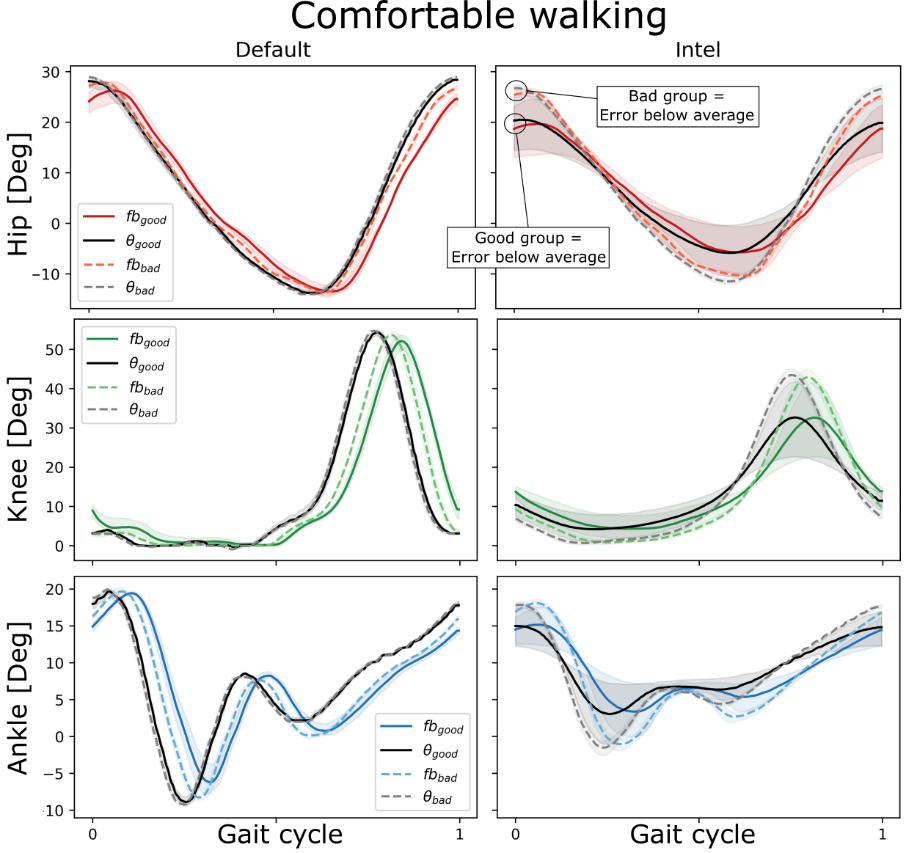


**Fig 6. Average hip, knee, and ankle trajectories of all subjects during comfortable walking in the default and intelligent control modes.** From the hip difference score ($\Delta e_{rms,h}$), its average value is used to separate subjects into two groups: good group with score below the average, and bad group with score above the average.

## **Interaction torque**

The interaction torque indicating the force between the human (wearer) and exoskeleton is measured and an example of the ankle torque from multiple subjects is shown in **Fig. 7**.

Root-mean-square error of the interaction torque is also calculated here:

$$\tau_{rms, j}= \sqrt{\frac{1}{N_{c}}\sum_{c=0}^{N_{c}} (\frac{1}{T_{c}}\sum_{t=0}^{T_{c}} \left( \tau_{j}^{c}\left[ t \right] \right)^{2})}.$$

The different score of interaction torque is also defined as well:

$$\Delta\tau_{rms,j}=\tau_{rms, j}^{I}-\tau_{rms, j}^{D}$$

An example of the different score is shown in **Fig 8**. The graph shows the same downward of the score which means that the intelligent control mode performs better than the default control mode when the walking frequency increases.


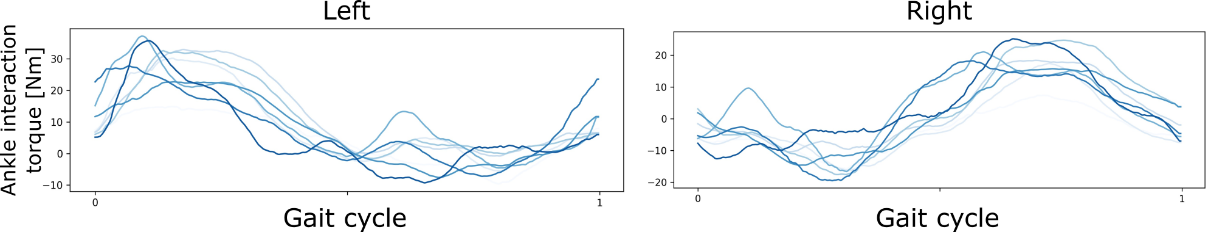


**Fig 7. An example of average interaction torque at ankle joint of both legs for all subjects.** Dimer line represents the first subject, while darker line represents the later subject.


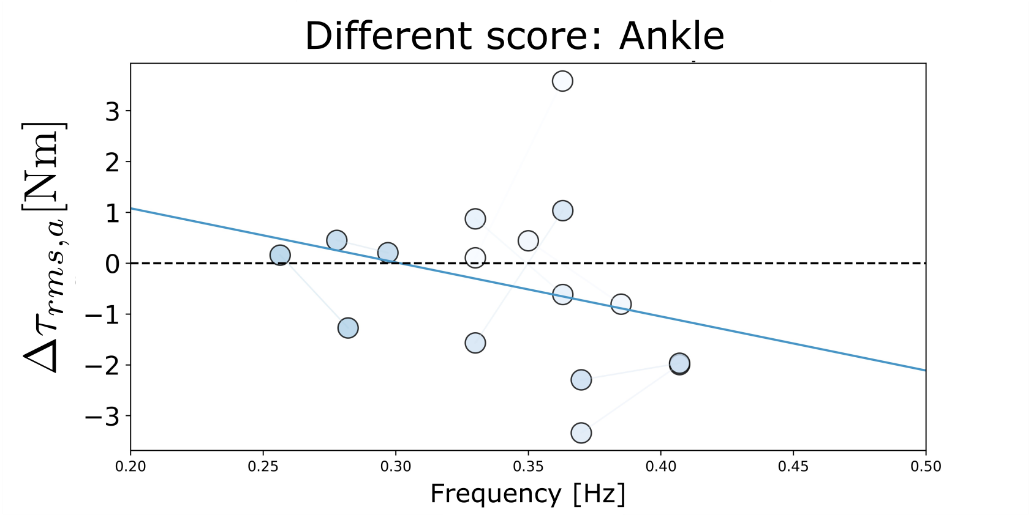


**Fig 8. Different score of interaction torque of ankle (**$\boldsymbol{\Delta}\boldsymbol{\tau}_{\boldsymbol{rms,a}}$**)**

# **References**

1. Bortole M, Venkatakrishnan A, Zhu F, Moreno JC, Francisco GE, Pons JL, et al. The H2 robotic exoskeleton for gait rehabilitation after stroke: early findings from a clinical study. Journal of NeuroEngineering and Rehabilitation. 2015;12(1):54. doi: 10.1186/s12984-015-0048-y.

2. Gil-Agudo A, Del Ama-Espinosa AJ, Lozano-Berrio V, Fernández-López A, Megía García-Carpintero A, Benito-Penalva J, et al. [Robot therapy with the H2 exoskeleton for gait rehabilitation in patients with incomplete spinal cord injry. A clinical experience]. Rehabilitacion. 2020;54(2):87-95. Epub 2020/05/07. doi: 10.1016/j.rh.2019.10.004. PubMed PMID: 32370833.

3. Srisuchinnawong A, Akkawutvanich C, Manoonpong P. Adaptive Modular Neural Control for Online Gait Synchronization and Adaptation of an Assistive Lower-Limb Exoskeleton. IEEE transactions on neural networks and learning systems. 2023;Pp. Epub 2023/04/08. doi: 10.1109/tnnls.2023.3263044. PubMed PMID: 37027271.

4. Akkawutvanich C, Manoonpong P. Personalized Symmetrical and Asymmetrical Gait Generation of a Lower Limb Exoskeleton. IEEE Transactions on Industrial Informatics. 2023;19(9):9798-808. doi: 10.1109/TII.2023.3234619.
