# Supplementary material for: Comprehensive multi-metric analysis of user experience and performance in adaptive and non-adaptive lower-limb exoskeletons
Source: PLoS One. 2025 Jan 9;20(1):e0313593. doi: 10.1371/journal.pone.0313593 (PMC11717227; doi:10.1371/journal.pone.0313593)
Supplement: S2 File — (DOCX) [file pone.0313593.s004.docx]

**S2 File. The speed of Exo-H3**

**Table 1.** The default 1-10 speed levels of Exo-H3. Each speed level produces specific gait cycle [s] for hip's, knee's, and ankle's joint profiles.

| **Speed level** | **Gait cycle time [s]** | **Swing frequency [Hz]** |
| --- | --- | --- |
| 1 | 4.5 | 0.222 |
| 2 | 4.2 | 0.238 |
| 3 | 3.9 | 0.256 |
| 4 | 3.6 | 0.277 |
| 5 | 3.3 | 0.303 |
| 6 | 3.0 | 0.333 |
| 7 | 2.7 | 0.370 |
| 8 | 2.4 | 0.417 |
| 9 | 2.1 | 0.476 |
| 10 | 1.8 | 0.556 |

**Table 2**. The conversion table to change swing frequency [Hz] to speed level. We define each frequency range based on gait cycle time [s] Table 1.

| **Swing frequency [Hz]** | **Gait cycle time [s]** | **Speed level** |
| --- | --- | --- |
| $\leq0.222$ | $\geq4.5$ | 1 |
| $>0.222- \leq0.238$ | $<4.5- \geq4.2$ | 2 |
| $>0.238- \leq0.256$ | $<4.2- \geq3.9$ | 3 |
| $>0.256- \leq0.277$ | $<3.9- \geq3.6$ | 4 |
| $>0.277- \leq0.303$ | $<3.6- \geq3.3$ | 5 |
| $>0.303- \leq0.333$ | $<3.3- \geq3.0$ | 6 |
| $>0.333- \leq0.370$ | $<3.0- \geq2.7$ | 7 |
| $>0.370- \leq0.417$ | $<2.7- \geq2.4$ | 8 |
| $>0.417- \leq0.476$ | $<2.4- \geq2.1$ | 9 |
| $>0.476$ | $<2.1$ | 10 |

**Table 3.** The subjects' walking information. Comfortable speed is found by Table 1.High speeds are found by Table 2.

| **Subject ID** | **Height [cm.]** | **Weight [Kg.]** | **Age [years]** | **Comfortable**  **freq. [Hz] / speed** | **High**  **freq. [Hz] / speed** |
| --- | --- | --- | --- | --- | --- |
| 1 | 170 | 47 | - | 0.333 / 6 | 0.363 / 7 |
| 2 | 166 | 51 | - | 0.350 / 7 | 0.385 / 8 |
| 3 | 167 | 56 | - | 0.333 / 6 | 0.363 / 7 |
| 4 | 177 | 73 | 30 | 0.370 / 7 | 0.407 / 8 |
| 5 | 170 | 70 | 30 | 0.333 / 6 | 0.363 / 7 |
| 6 | 164 | 52 | 27 | 0.370 / 7 | 0.407 / 8 |
| 7 | 160 | 47 | 29 | 0.278 / 4 | 0.297 / 5 |
| 8 | 160 | 68 | 28 | 0.256 / 3 | 0.282 / 5 |
