# Supplementary figures and images for: Comprehensive multi-metric analysis of user experience and performance in adaptive and non-adaptive lower-limb exoskeletons
Source: PLoS One. 2025 Jan 9;20(1):e0313593. doi: 10.1371/journal.pone.0313593 (PMC11717227; doi:10.1371/journal.pone.0313593)

**
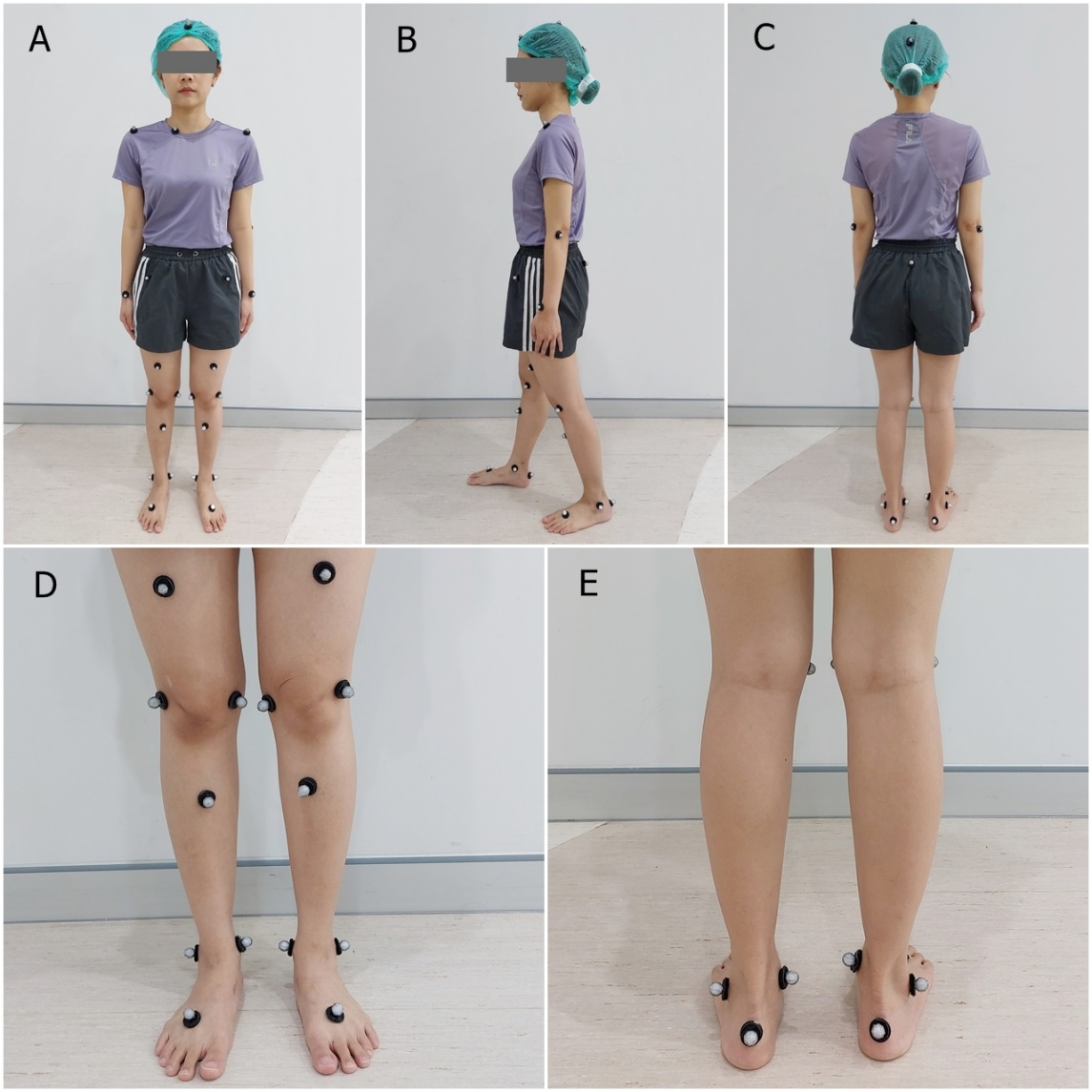
S3 File. Reflective markers based on modified Helen Hayes**

Supplement: S3 File — (DOCX) [file pone.0313593.s005.docx]
