# Supplementary material for: Comprehensive multi-metric analysis of user experience and performance in adaptive and non-adaptive lower-limb exoskeletons
Source: PLoS One. 2025 Jan 9;20(1):e0313593. doi: 10.1371/journal.pone.0313593 (PMC11717227; doi:10.1371/journal.pone.0313593)
Supplement: S1 Table — (DOCX) [file pone.0313593.s006.docx]

**S1 Table. Characteristic of the participants*.*** Sequence AB = the default mode followed by the intelligent mode, BA = the intelligent mode followed by the default mode, BMI = body mass index

| **ID** | **Group** | **Sequence** | **Sex** | **Age**  **(year)** | **Weight** | **Height** | **BMI** | **Shoe size** | **Underlying disease** | **Education** |
| --- | --- | --- | --- | --- | --- | --- | --- | --- | --- | --- |
| 1 | 1 | AB | M | 20 | 54 | 170 | 18.69 | 42 | No | high school |
| 2 | 1 | AB | M | 23 | 51 | 166 | 18.51 | 41 | No | bachelor’s degree |
| 3 | 2 | BA | F | 21 | 56 | 167 | 20.58 | 38 | No | bachelor’s degree |
| 4 | 2 | BA | M | 30 | 72 | 171 | 24.62 | 41 | No | bachelor’s degree |
| 5 | 2 | BA | F | 29 | 70 | 170 | 24.22 | 39 | No | bachelor’s degree |
| 6 | 1 | AB | F | 27 | 52 | 164 | 19.33 | 39 | No | bachelor’s degree |
| 7 | 1 | AB | F | 29 | 48 | 160 | 18.75 | 37 | No | bachelor’s degree |
| 8 | 2 | BA | F | 28 | 63 | 160 | 24.61 | 39 | No | bachelor’s degree |
