# Supplementary material for: Comprehensive multi-metric analysis of user experience and performance in adaptive and non-adaptive lower-limb exoskeletons
Source: PLoS One. 2025 Jan 9;20(1):e0313593. doi: 10.1371/journal.pone.0313593 (PMC11717227; doi:10.1371/journal.pone.0313593)
Supplement: S2 Table — (DOCX) [file pone.0313593.s007.docx]

**S2 Table.** **The comparison of lower limb kinematics between groups during walking without the exoskeleton***.* SD = standard deviation, + = pelvic obliquity up, pelvic tilt anterior, pelvic internal rotation, hip adduction, hip flexion, hip internal rotation, knee varus, knee flexion, knee internal rotation, ankle varus, ankle dorsiflexion, ankle internal rotation, internal foot progression, - = pelvic obliquity down, pelvic tilt posterior, pelvic external rotation, hip abduction, hip extension, hip external rotation, knee valgus, knee extension, knee external rotation, ankle valgus, ankle plantar flexion, ankle external rotation, external rotation foot progression, *P-value <0.05, P-value from Independent t-test, ^a^P-value from Mann-Whitney U test.

| **Kinematics (degrees)** | **Group 1**  (n=4) | | **Group 2**  (n=4) | | **P-value** |
| --- | --- | --- | --- | --- | --- |
|  | Mean | SD | Mean | SD |  |
| **Pelvic** |  |  |  |  |  |
| **Pelvic Obliquity Up/ Down** |  |  |  |  |  |
| Heel strike | 0.55 | 0.31 | 0.35 | 1.29 | 0.248^a^ |
| Mid-stance | 2.35 | 1.22 | 2.45 | 0.79 | 1.000^a^ |
| Terminal stance | 0.15 | 0.43 | -0.08 | 1.13 | 0.773^a^ |
| Mid swing | -1.39 | 1.15 | -0.61 | 1.03 | 0.387^a^ |
| **Pelvic Anterior/ Posterior Tilt** |  |  |  |  |  |
| Heel strike | 2.65 | 3.38 | 7.42 | 4.15 | 0.083^a^ |
| Mid-stance | 2.59 | 3.99 | 6.87 | 4.05 | 0.149^a^ |
| Terminal stance | 3.19 | 3.59 | 7.67 | 3.59 | 0.083^a^ |
| Mid swing | 2.82 | 3.93 | 7.04 | 3.93 | 0.149^a^ |
| **Pelvic Internal/ External Rotation** |  |  |  |  |  |
| Heel strike | 2.68 | 2.61 | 4.44 | 1.94 | 0.248^a^ |
| Mid-stance | 1.59 | 1.57 | 2.43 | 1.05 | 0.773^a^ |
| Terminal stance | -1.35 | 1.19 | -1.96 | 1.01 | 0.387^a^ |
| Mid swing | -1.29 | 0.93 | -1.29 | 0.69 | 0.564^a^ |
| **Hip** |  |  |  |  |  |
| **Hip Adduction/Abduction** |  |  |  |  |  |
| Heel strike | 1.26 | 3.72 | 0.86 | 0.74 | 1.000^a^ |
| Mid-stance | 5.73 | 3.23 | 7.19 | 2.78 | 0.564^a^ |
| Terminal stance | 3.84 | 3.23 | 5.11 | 2.64 | 0.564^a^ |
| Mid swing | -1.18 | 3.25 | -2.18 | 3.31 | 0.564^a^ |
| **Hip Flexion/ Extension** |  |  |  |  |  |
| Heel strike | 24.11 | 6.84 | 34.53 | 5.01 | 0.049* |
| Mid-stance | 9.33 | 5.18 | 19.16 | 4.84 | 0.043*^a^ |
| Terminal stance | -3.99 | 5.99 | 3.54 | 5.79 | 0.083^a^ |
| Mid swing | 28.40 | 10.29 | 34.53 | 4.68 | 0.321 |
| **Hip Internal/ External rotation** |  |  |  |  |  |
| Heel strike | 2.08 | 9.74 | 2.34 | 8.59 | 1.000^a^ |
| Mid-stance | 0.70 | 11.39 | -0.37 | 10.41 | 0.564^a^ |
| Terminal stance | -0.83 | 11.19 | -1.21 | 10.89 | 0.773^a^ |
| Mid swing | -1.02 | 10.33 | -3.77 | 8.71 | 0.564^a^ |
| **Knee** |  |  |  |  |  |
| **Knee Varus/ Valgus** |  |  |  |  |  |
| Heel strike | 0.55 | 3.70 | -1.32 | 2.07 | 0.387^a^ |
| Mid-stance | 1.09 | 3.55 | -1.11 | 2.16 | 0.387^a^ |
| Terminal stance | 0.82 | 3.41 | -2.47 | 1.09 | 0.083^a^ |
| Mid swing | -0.32 | 5.21 | -0.16 | 3.52 | 0.773^a^ |
| **Knee Flexion/ Extension** |  |  |  |  |  |
| Heel strike | 7.63 | 3.59 | 13.21 | 4.19 | 0.089 |
| Mid-stance | 10.95 | 4.68 | 18.00 | 6.57 | 0.131 |
| Terminal stance | 11.07 | 7.31 | 14.72 | 3.96 | 0.387^a^ |
| Mid swing | 46.56 | 19.54 | 56.75 | 1.51 | 0.339 |
| **Knee IR/ER** |  |  |  |  |  |
| Heel strike | -15.89 | 8.89 | -21.66 | 15.91 | 0.564^a^ |
| Mid-stance | -12.21 | 9.67 | -16.57 | 17.85 | 1.000^a^ |
| Terminal stance | -9.86 | 10.69 | -12.31 | 15.89 | 1.000^a^ |
| Mid swing | -20.94 | 11.09 | -24.10 | 11.67 | 0.564^b^ |
| **Ankle** |  |  |  |  |  |
| **Ankle Varus/Valgus** |  |  |  |  |  |
| Heel strike | -4.66 | 3.36 | -7.27 | 9.00 | 0.564^a^ |
| Mid-stance | -7.46 | 3.13 | -10.43 | 11.34 | 0.387^a^ |
| Terminal stance | -7.35 | 3.64 | -11.41 | 14.54 | 0.564^a^ |
| Mid swing | -5.48 | 3.23 | -7.56 | 10.94 | 0.387^a^ |
| **Ankle DF/PF** |  |  |  |  |  |
| Heel strike | -2.80 | 3.33 | -2.15 | 6.08 | 0.773^a^ |
| Mid-stance | 3.39 | 2.96 | 4.42 | 6.11 | 0.773^a^ |
| Terminal stance | 10.99 | 2.40 | 11.75 | 4.66 | 0.783 |
| Mid swing | 2.32 | 3.09 | 0.85 | 3.84 | 0.773^a^ |
| **Ankle IR/ER** |  |  |  |  |  |
| Heel strike | 0.72 | 1.88 | 1.76 | 8.67 | 0.773^a^ |
| Mid-stance | 1.22 | 2.44 | 1.41 | 7.79 | 0.773^a^ |
| Terminal stance | 2.34 | 2.07 | 2.84 | 7.01 | 1.000^a^ |
| Mid swing | 2.83 | 1.45 | 4.95 | 8.82 | 1.000^a^ |
| **Foot Progression IR/ER** |  |  |  |  |  |
| Heel strike | -10.07 | 3.53 | -10.39 | 3.60 | 0.901 |
| Mid-stance | -7.96 | 3.15 | -8.16 | 2.28 | 0.921 |
| Terminal stance | -9.89 | 2.06 | -10.01 | 2.24 | 0.936 |
| Mid swing | -20.01 | 6.32 | -22.02 | 9.33 | 0.733 |
