# Supplementary material for: Comprehensive multi-metric analysis of user experience and performance in adaptive and non-adaptive lower-limb exoskeletons
Source: PLoS One. 2025 Jan 9;20(1):e0313593. doi: 10.1371/journal.pone.0313593 (PMC11717227; doi:10.1371/journal.pone.0313593)
Supplement: S3 Table — (DOCX) [file pone.0313593.s008.docx]

**S3 Table. The comparison of muscle activity (%RMS) between groups during walking without the exoskeleton***.* SD = standard deviation, *P-value <0.05, P-value from Independent t-test, ^a^P-value from Mann-Whitney U test

| **Muscle activity (V)** | **Group 1** | | **Group 2** | | **P-value** |
| --- | --- | --- | --- | --- | --- |
|  | Mean | SD | Mean | SD |  |
| Rt. gluteus maximus | 11.94 | 5.34 | 46.05 | 54.75 | 0.149^a^ |
| Lt. gluteus maximus | 19.68 | 15.89 | 68.66 | 62.59 | 0.083^a^ |
| Rt. Tibialis Anterior | 19.59 | 8.42 | 26.32 | 11.13 | 0.372 |
| Lt. tibialis anterior | 13.76 | 5.41 | 21.59 | 6.23 | 0.106 |
| Rt. gastrocnemius | 42.01 | 27.82 | 76.02 | 31.72 | 0.083^a^ |
| Lt. gastrocnemius | 25.67 | 14.21 | 47.25 | 27.18 | 0.248^a^ |
| Rt. rectus femoris | 9.62 | 5.83 | 28.16 | 18.28 | 0.083^a^ |
| Lt. rectus femoris | 11.84 | 6.28 | 14.33 | 11.87 | 1.000^a^ |
| Rt. biceps femoris | 8.55 | 4.01 | 23.58 | 11.39 | 0.047* |
| Lt. biceps femoris | 8.05 | 4.00 | 18.79 | 12.51 | 0.149^a^ |
