# Supplementary material for: Comprehensive multi-metric analysis of user experience and performance in adaptive and non-adaptive lower-limb exoskeletons
Source: PLoS One. 2025 Jan 9;20(1):e0313593. doi: 10.1371/journal.pone.0313593 (PMC11717227; doi:10.1371/journal.pone.0313593)
Supplement: Attachment — Submitted filename: Comments.pdf [file pone.0313593.s010.pdf]

The manuscript entitled “Comprehensive Multi-Metric Analysis of User Experience and Performance in Adaptive and Non-adaptive Lower-limbExoskeletons” evaluates the user experience and performance of adaptive versus non-adaptive lower-limb exoskeletons through a multi-metric analysis involving gait, muscle activity, oxygen consumption, and comfort. Using a crossover design with healthy adults, findings revealed that adaptive exoskeletons, utilizing central pattern generator-based controls, exhibited lower velocity and stride lengths but offered advantages in specific kinematic adjustments and reduced oxygen consumption, suggesting a need for further refinement in exoskeleton design to enhance user comfort and performance.The study is well-developed and the results are intriguing. However, the following concerns need to be addressed:

1. **Methodological Rigor**: The study design is a crossover trial; however, can you clarify if there were any washout periods between conditions to prevent carry-over effects from one exoskeleton mode to the other? Additionally, how was the sample size determined, and could you provide more details on the statistical power analysis?
2. **Control Conditions and Variables**: In comparing adaptive and non-adaptive exoskeletons, how did you control for external variables such as participants' physical condition and previous experience with similar devices? How might these factors have influenced the outcomes?
3. **Results Interpretation**: You reported significant differences in stride length and velocity between the two modes. How do these differences translate into practical benefits or drawbacks for users in real-world scenarios?
4. **Technical Specifications**: Could you provide more detailed specifications of the adaptive control mechanisms in the exoskeletons? How do these mechanisms adapt in real-time to the user's gait dynamics, and what algorithms support this adaptability?
5. **Biomechanical Measurements**: What methods were used to measure kinematics and muscle activity, and how did you ensure the accuracy and reliability of these measurements, especially given the potential for interference from the exoskeleton itself?
6. **User Experience**: The study measures user comfort and performance scores; however, can you elaborate on how these were quantified? What specific aspects of user experience were evaluated, and how were these aspects chosen?
7. **Future Research Directions**: Based on the findings, what specific areas of exoskeleton design do you think require further investigation? Are there particular challenges that emerged from this study that future research needs to address?
8. **Comparison with Existing Technologies**: How does the performance of the adaptive exoskeleton compare with current state-of-the-art devices in terms of user experience and biomechanical performance? Are there competitive technologies that offer better results?
9. **References**: The following refeercnes are highly recommended toa dded to the paper to improve its quality and state of the arts. (10.1109/TNSRE.2021.3136088), (10.1109/ACCESS.2023.3325211), and (10.3390/s23136103).
