# Supplementary material for: Comprehensive multi-metric analysis of user experience and performance in adaptive and non-adaptive lower-limb exoskeletons
Source: PLoS One. 2025 Jan 9;20(1):e0313593. doi: 10.1371/journal.pone.0313593 (PMC11717227; doi:10.1371/journal.pone.0313593)
Supplement: Attachment — Submitted filename: Response to reviewers_Plos One_Exoskeleton.docx [file pone.0313593.s011.docx]

**Response to reviewers’ comments**

**Reviewer #1: The manuscript entitled “Comprehensive Multi-Metric Analysis of User Experience and Performance in Adaptive and Non-adaptive Lower-limb Exoskeletons”** evaluates the user experience and performance of adaptive versus non-adaptive lower-limb exoskeletons through a multi-metric analysis involving gait, muscle activity, oxygen consumption, and comfort. Using a crossover design with healthy adults, findings revealed that adaptive exoskeletons, utilizing central pattern generator-based controls, exhibited lower velocity and stride lengths but offered advantages in specific kinematic adjustments and reduced oxygen consumption, suggesting a need for further refinement in exoskeleton design to enhance user comfort and performance. The study is well-developed and the results are intriguing. **However, the attached concerns need to be addressed.**

**1. Methodological Rigor: The study design is a crossover trial; however, can you clarify if there were any washout periods between conditions to prevent carry-over effects from one exoskeleton mode to the other? Additionally, how was the sample size determined, and could you provide more details on the statistical power analysis?**

**Answer for wash out periods:**

Thank you for your questions. Regarding the washout period, we ensured that each participant had a minimum of 10 minutes of rest as a washout period before moving on to the next exoskeleton mode. The transition to the next mode only occurred when the participant felt comfortable and ready to proceed. This approach aligns with the study of Hybart et al. (2023), where the authors stated, "Between each condition, the participants rest as needed." Based on this reference, we believe the washout period we implemented is sufficient to prevent carry-over effects between conditions.

**Revision:**

I have revised the content in the manuscript line 164-165

From

“A 10-minute resting period was implemented between each intervention to reduce the carryover effect.”

To

“The participants had a minimum of 10 minutes of rest [23], or more if needed, between each intervention to minimize carryover effects [24].”

**Answer for sample size calculation:**

For sample size calculation, we followed the guidelines provided by Assoc. Prof. Cameron Hurst from Chulalongkorn University. We used the sample size calculation tool available at this website <https://www2.ccrb.cuhk.edu.hk/stat/mean/tsmc_sup.htm> After filling the required parameters specifically, a mean difference of 6.8 degrees from the study of Guzik et al.(2020), a standard deviation (SD) of 7.6, and a margin of 5 degrees—the calculated total sample size was 8 participants for the crossover design.

**Revision:**

I have revised the content of sample size calculation in the manuscript line 227-232

From

“Sample size estimation was performed based on evidence from the mean and standard deviations of peak value of knee extension of the unaffected leg in stroke patients with Knee Ankle Foot Orthosis (KAFO) (-0.8±7.6 degrees) compared to those without KAFO (0.2±7.2 degrees). The study used a type I error (alpha) of 0.05 and power of 0.8. Therefore, a sample size was 4 participants per group.”

To

“Sample size estimation was conducted using a sample size calculator for crossover design (https://www2.ccrb.cuhk.edu.hk/ stat/mean/tsmc_sup.htm). The estimation was based on a mean difference of 6.8 degrees [29], a standard deviation (SD) of 7.6 degrees, and a margin of 5 degrees. The study used a type I error (alpha) of 0.05 and power of 0.8. Therefore, the total sample size required for the study was 8 participants.”

Reference:

R. L. Hybart and D. P. Ferris, "Neuromechanical Adaptation to Walking With Electromechanical Ankle Exoskeletons Under Proportional Myoelectric Control," in IEEE Open Journal of Engineering in Medicine and Biology, vol. 4, pp. 119-128, 2023, doi: 10.1109/OJEMB.2023.3288469

Guzik A, Drużbicki M, Wolan-Nieroda A, Turolla A, Kiper P. Estimating Minimal Clinically Important Differences for Knee Range of Motion after Stroke. J Clin Med. 2020 Oct 15;9(10):3305. doi: 10.3390/jcm9103305. PMID: 33076214; PMCID: PMC7602397.

**2. Control Conditions and Variables: In comparing adaptive and non-adaptive exoskeletons, how did you control for external variables such as participants' physical condition and previous experience with similar devices? How might these factors have influenced the outcomes?**

**Answer:**

The research team used strict inclusion criteria to select participants who were healthy, had no walking impairments, and had no prior experience using similar devices. On the day of the experiment, each participant was trained under expert supervision to walk with the exoskeleton until they were confident and able to walk independently. Additionally, this study employed a crossover design, which compared outcomes within the same subjects, thereby ensuring that prognostic factors were comparable and minimizing the influence of external variables.

**3. Results Interpretation: You reported significant differences in stride length and velocity**

**between the two modes. How do these differences translate into practical benefits or**

**drawbacks for users in real-world scenarios?**

**Answer:**

From our data, the intelligence mode diminished the velocity approximately 9 cm/s and stride length by 22-23 cm when compared to the default exoskeleton at both speeds. The benefits for the real world are probably more stability by controlling center of gravity (Qiu S, 2023), and facilitates comfortability and performance, especially at high speed. The drawback of the intelligence setting is slowly forward moving.

The explanations are the conflict between intrinsic and forced walking styles occurred when the subjects are forced to walk with fixed speed in the default mode. Our control algorithm can adapt to different users which can be seen from the results showing different stride lengths and velocities. Normally, each subject has his/her own intrinsic preferred swing frequency (which can be indirectly translated into the stride length) and the walking speed (velocity) when the subjects walk freely without wearing the exoskeleton. When the subjects are forced to walk in the default mode where the speed is fixed and cannot be altered during walking, the conflict between intrinsic and forced walking styles might occur. Our control algorithm plays an important role to mitigate this conflict by allowing the change to be occurred according to the subjects’ intention when the exoskeleton forces them out of their comfortable walking style (stride length and velocity in this case). For example, one subject may walk naturally fast, but he/she feels insecure while assisting by the exoskeleton at the high speed and preferred to tone it down which is allowed by the algorithm. However, when both things (the exoskeleton and the subject) adapt simultaneously for each other, this seems to be difficult in some cases comparing to human adaptation only (in case of default mode, various speeds).

References

1. Qiu, S., Pei, Z., Wang, C. et al. Systematic Review on Wearable Lower Extremity Robotic Exoskeletons for Assisted Locomotion. J Bionic Eng 20, 436–469 (2023). <https://doi.org/10.1007/s42235-022-00289-8>

**Revision:** Revision was made in the Discussion part

From

“Regarding to this study, walking with either the default or intelligent-mode exoskeleton significantly decelerated velocity and cadence when compared to normal walking at both speed (p<0.001). Moreover, the default setting compromised the percentage of left swing but increased step width in contrast to walking without the exoskeleton.”

To

“**Line 417-421:** Regarding to this study, walking with either the default or intelligent-mode exoskeleton significantly decelerated velocity and cadence when compared to normal walking at both speed (p<0.001). Moreover, the default setting compromised the percentage of left swing but increased step width in contrast to walking without the exoskeleton”.

“**Line 431-440:** The explanations are the conflict between intrinsic and forced walking styles occurred when the subjects are forced to walk with fixed speed in the default mode. Our control algorithm plays an important role to mitigate this conflict by allowing the change to be occurred according to the subjects’ intention when the exoskeleton forces them out of their comfortable walking style (stride length and velocity in this case). However, the exoskeleton-subject adaptation seems to be difficult in some cases comparing to the default mode at various speeds.The intelligence mode diminished the velocity approximately 9 cm/s and stride length by 22-23 cm when compared to the default exoskeleton at both speeds. The benefits of this algorithm for the real world are probably more stability by controlling center of gravity [24], and facilitates comfortability and performance, especially walking at high speed (Table 4). The drawback of the intelligence setting is slowly forward moving.”

**4. Technical Specifications: Could you provide more detailed specifications of the adaptive control mechanisms in the exoskeletons? How do these mechanisms adapt in real-time to the user's gait dynamics, and what algorithms support this adaptability?**

**Answer:**

Thank you so much for your suggestion. The detailed specification of adaptive control mechanisms and its effects towards user’s kinematics has already provided in the supplementary file (S1 File) as following

For the default control mode (Fig 2A), joint position patterns (θ ̅) of hip, knee, and ankle for both legs are obtained from the company. These joint patterns are average profiles collected from European subjects. When assist, our mid-level control algorithm samples the joint profile and directly sends each joint angle data (θ) in an appropriate time to moderate a swing speed without any feedback information (open-loop control). The default control mechanism is designed for gait generation without adaptation during locomotion.

For the intelligent control mode [3], it performs as closed-loop control based on six neural control modules (modules 1-6, Fig 2B.) with interconnected feedback. Each module is designed to interact with other modules uniquely. The hip and knee modules share their feedback pathways (δ) to all the other modules, while ankle modules only receive adaptation signals from the others. This coupling structure with bidirectional hip and knee feedback pathways is adopted to ensure a 90-degree phase difference between the left and right legs. The joint module (e.g., left hip module as an example in Fig 2B.) uses tracking loop concept where the algorithm adjusts its previous joint control signal to be suitable for a wearer in a current step. The module consists of six key components: phase generator, pattern generator, transformation equation (forward dynamics), time constant, gradient-based adaptation, and coupled cooperative primitives (CCP) adaptation. The phase generator creates rhythmic phase signals (C_1, C_2) based on a central pattern generator (CPG) concept. Those signals then activate the pattern generator module to produce a reference joint position angle at each time step (f) in this case for the left hip whose total profile/pattern is the joint position profile (θ ̅) from the factory that has been previously learned and remembered by the module via weights of neural networks. The transformation eq. is then introduced with time constant block to create joint compliance on the system by making changes (τθ ̇) on top of the reference joint position angle (f). An adaptation part comes from changing tracking error (e= θ-θ^*) into two forms to be used by the gradient-based adaptation and the CCP adaptation modules. The former uses the error to adjust phase of the control signal, while the latter regulates the joint compliant path. Note that the phase gradient adaptation signal (δ) does not come only from the working module itself (δ_LH), but also from the other modules (δ_(other modules)) as mentioned earlier on the pathways. All in all, the intelligent control mechanism is designed for gait generation and adaptation in response to the tracking error during locomotion.

The algorithms support its adaptability was elaborated as Fig. 2 in the supplementary file (S1 File) as

**Fig 2.** (A) Block diagram of default control algorithm in this study. The factory joint position profile is used to drive the exoskeleton with adjustable swing speed. (B) Block diagram of intelligent control algorithm in this study. It is Adaptive Modular Neural Control (AMNC) for online gait synchronization. RH/LH = Right/Left hip, RK/LK = Right/Left knee, and RA/LA = Right/left ankle.

**5. Biomechanical Measurements: What methods were used to measure kinematics and muscle activity, and how did you ensure the accuracy and reliability of these measurements, especially given the potential for interference from the exoskeleton itself?**

**Answer:**

To measure kinematics and spatio-temporal parameters, we employed a highly reliable eight-camera motion analysis system from Motion Analysis Corporation (Santa Rosa, CA, USA), which is widely used in research for its accuracy and reliability. Muscle activity was assessed using surface electromyography (sEMG) with ProEMG software operating at 2000 Hz, alongside a Myon 320 wireless EMG system (Myon AG, Schwarzenberg, Switzerland), both of which are well-regarded in the field for their precision and have been utilized in numerous studies. To ensure the accuracy and reliability of our measurements, we performed calibration of these systems before each data collection session. This crucial step accounted for any potential interference from the exoskeleton and maintained the integrity of the collected data. We hope this clarifies the methods used and the steps taken to ensure the reliability of our measurements.

**Revision:**

I have revised the manuscript by adding the sentence, 'To ensure the accuracy and reliability of our measurements, we performed calibration of these systems before each data collection session,' to lines 183-184.

**6. User Experience: The study measures user comfort and performance scores; however, can you elaborate on how these were quantified? What specific aspects of user experience were evaluated, and how were these aspects chosen?**

**Answer:**

Thank you so much for your queries. The user experience was evaluated using an exoskeletal performance questionnaire that comprised two domains: pain (while walking, rising from sitting, and standing) and difficulty (in rising from sitting, standing, and walking for distance). These aspects of user experience were selected based on the anticipated activities that users would engage in on the day of the study, such as rising from sitting, standing, and walking with the exoskeleton. The questions were chosen in advance to ensure their relevance to these activities, in accordance with our protocol focused on gait measurement. This approach allowed us to capture the most relevant experiences of discomfort (pain) and difficulty, providing a comprehensive assessment of the user experience with the exoskeleton.

The exoskeletal performance questionnaire comprised 2 domains; pain (walking, rising from sitting, standing) and difficulty (rising from sitting, standing, forward bending, walking, walking for distance). Each question was rated by respondents ranged from 1 (minimal issues) to 5 (significant problems). The total performance score was defined as best (8 points), good (9-23 points), moderate (24 points), poor (25-39 points), and worst (40 points).

Also, the comfort assessment reference (J. F. Knight, C. Baber, A. Schwirtz and H. W. Bristow, "The comfort assessment of wearable computers," Proceedings. Sixth International Symposium on Wearable Computers, Seattle, WA, USA, 2002, pp. 65-72, doi: 10.1109/ISWC.2002.1167220) has been cited.

**Revision:** The revision was performed, line 213-226

From

“For the wearer's opinion to the device, their perceived change, emotion on self-image, anxiety on security, harm or painful, attachment, and movement were evaluated as the comfort score. Each item rated from a 1 (the least problem) to 5 (the most problem). Sum of total score was 30, and categorized as very comfortable (1-6 points), comfortable (7-17 points), moderately comfortable (18 points), uncomfortable (19-29 points), and uncomfortable at all (30 points).

The exoskeletal performance questionnaire comprised 2 domains; pain (walking, rising from sitting, standing) and difficulty (rising from sitting, standing, walking for distance). Respondents rated all 11 questions ranged from 1 (minimal issues) to 5 (significant problems). The total performance score was designated as the best performance (1-11 points), good performance (12-13 points), moderate performance (33 point), poor performance (34-54 points), and the worst performance (55 points).”

To

“For the wearer's opinion to the device, their perceived change, emotion on self-image, anxiety on security, harm or painful, attachment, and movement were evaluated as the comfort score[25]. Each item rated from a 1 (the least problem) to 5 (the most problem). Sum of total score was 30, and categorized as very comfortable (1-6 points), comfortable (7-17 points), moderately comfortable (18 points), uncomfortable (19-29 points), and uncomfortable at all (30 points).

User experience was evaluated using an exoskeletal performance questionnaire that comprised two domains: pain (while walking, rising from sitting, and standing) and difficulty (in rising from sitting, standing, forward bending, walking, and walking for distance). In accordance with our protocol focused on gait measurement, this approach captured the most relevant experiences of discomfort (pain) and difficulty, providing a comprehensive assessment of the user experience with the exoskeleton. Respondents rated all 8 questions ranged from 1 (minimal issues) to 5 (significant problems). The total performance score was designated as the best performance (8 points), good performance (9-23 points), moderate performance (24 points), poor performance (25-39 points), and the worst performance (40 points).”

**7. Future Research Directions: Based on the findings, what specific areas of exoskeleton design do you think require further investigation? Are there particular challenges that emerged from this study that future research needs to address?**

**Answer:**

Thank you so much for your questions. Besides interaction torque reduction by joint angle control adaptation, future research is suggested for both hardware and software. For the former, replacing the full-body exoskeleton to modular type e.g., knee exoskeleton may facilitate more flexible motion. For the latter, free-mode + central pattern generators (CPG) + radius basis function (RBF) is required in order to learn more human-like patterns (kinematics).

**Revision:** The conclusion is revised by adding this information line 481-491

From

“Adaptive exoskeleton as an intelligent mode improves muscle activity, oxygen consumption, user comfort and performance of the device. Further large studies are needed to refine the temporal-spatial, lower extremity kinematics, particularly hip-knee-ankle sagittal motions, to synchronize body and exoskeleton mobility.”

To

“Adaptive exoskeleton as an intelligent mode improves muscle activity, oxygen consumption, user comfort and performance of the device. Further large studies are needed to refine the temporal-spatial, lower extremity kinematics, particularly hip-knee-ankle sagittal motions, to synchronize body and exoskeleton mobility. Besides interaction torque reduction by joint angle control adaptation, future research is suggested for both hardware and software. For the former, replacing the full-body exoskeleton to modular type e.g., knee exoskeleton may facilitate more flexible motion, and various hip-knee-ankle module combination. For the latter, free-mode or zero-torque control is required in order to learn more human-like patterns (kinematics). Also, applying CPG and radius basis function (RBF) to learn different frequencies and generate different shape/pattern will allow more changes in frequency and joint angle pattern.”

**8. Comparison with Existing Technologies: How does the performance of the adaptive exoskeleton compare with current state-of-the-art devices in terms of user experience and**

**biomechanical performance? Are there competitive technologies that offer better results?**

**Answer:**

Thank you so much for your suggestion and question.

In many commercialized exoskeletons such as ReWalk, Ekso, etc., they usually adopt the basic joint trajectory tracking control as their default control (Li WZ, 2021). The adaptive control, as in our case, is considered to be more advanced and unique for a wearer. Besides, compared to an exoskeleton from Cyberdyne using muscle signal as a trigger, its main purpose aims to create a gait movement with a body-supported device rather than solely assist a wearer energetically as for our Exo-H3 exoskeleton.

The amounts of gait parameter changes from our results were comparable with the other multi-joint adaptive technologies. Our average baseline gait parameters demonstrated velocity 100 cm/s, stride length 1.2 m, and stance phase 61%. The applying exoskeleton resulted in 58-75 cm/s velocity reduction, 0.3-29 cm shorter stride length, and 4-10% longer stance phase. Ekso Bionic demonstrated significant slower walking speed, shorter stride length, and longer double-limb support time than those without an exoskeleton (Swank C, 2019). Comparing with and without Ekso in 15 health adults, average gait parameters showed velocity 31.0 ± 0.4 vs 132.0 ± 1.6 cm/s, stride length 0.72 ± 0.14 vs 1.41 ± 0.12 m, and double support 4.5 ± 0.6 vs 1.7 ± 0.2 s.

Other potential multi-joint adaptive technologies are Ekso Bionics, Richmond, CA, USA (Swank C, 2019, Palermo AF, 2017)**,** and Hybrid Assistive Limb (HAL), Japan (Hassen M, 2018 and Qiu S, 2023*)*. Their performances are presented as following

- Ekso Bionic used for assisting gait in spinal cord injury (Edwards DJ, 2022) accelerated gait speed up to 18 ± 23 cm/s from the baseline 20 cm/s. Average Timed Up and Go Test (TUG) 26.4 s (quartile 17.3-53.0 s) and median 6-minute Walk Test (6MWT) 346.6 feet (quartile 219.5-711.5 feet).
- Hybrid Assistive Limb (HAL) supported walking ability in hemiplegic patients (Hassen M, 2018). This system enhanced joint motion of an affected limb, and feasible in 3 out of 5 participants. When compared non-HAL to synergy-based HAL gait, average cadence was reduced from 37.8 to 29.9 steps/min, and mean walking speed was slower from 57.4 to 16.2 cm/s. Hemiplegic participants spent more time in the stance phase in order to facilitate robot leg motion.

References

1. Li WZ, Cao GZ, Zhu AB. Review on Control Strategies for Lower Limb Rehabilitation Exoskeletons. IEEE Access. 2021;9:123040–60.
2. Swank C, Wang-Price S, Gao F, Almutairi S. Walking With a Robotic Exoskeleton Does Not Mimic Natural Gait: A Within-Subjects Study. JMIR Rehabil Assist Technol. 2019 Jan 14;6(1):e11023. doi: 10.2196/11023. PMID: 31344681; PMCID: PMC6682279.
3. Palermo AE, Maher JL, Baunsgaard CB, Nash MS. Clinician-Focused Overview of Bionic Exoskeleton Use After Spinal Cord Injury. Top Spinal Cord Inj Rehabil. 2017 Summer;23(3):234-244. doi: 10.1310/sci2303-234. PMID: 29339899; PMCID: PMC5562031.
4. Hassan M, Kadone H, Ueno T, Hada Y, Sankai Y, Suzuki K. Feasibility of Synergy-Based Exoskeleton Robot Control in Hemiplegia. IEEE Trans Neural Syst Rehabil Eng. 2018 Jun;26(6):1233-1242. doi: 10.1109/TNSRE.2018.2832657. PMID: 29877848.
5. Qiu, S., Pei, Z., Wang, C. et al. Systematic Review on Wearable Lower Extremity Robotic Exoskeletons for Assisted Locomotion. J Bionic Eng 20, 436–469 (2023). <https://doi.org/10.1007/s42235-022-00289-8>.
6. Edwards DJ, Forrest G, Cortes M, Weightman MM, Sadowsky C, Chang SH, Furman K, Bialek A, Prokup S, Carlow J, VanHiel L, Kemp L, Musick D, Campo M, Jayaraman A. Walking improvement in chronic incomplete spinal cord injury with exoskeleton robotic training (WISE): a randomized controlled trial. Spinal Cord. 2022 Jun;60(6):522-532. doi: 10.1038/s41393-022-00751-8. Epub 2022 Jan 29. PMID: 35094007; PMCID: PMC9209325.

**Revision:** Revision was made in the Discussion part, line 411-430

From

“The exoskeleton specifically changed gait parameters and walking habits in order to maintain the dynamic balance of the human body [14-16]. In work by Swank, et al.[17], the robotic exoskeleton (EKSO) had a slower walking speed, shorter steps, and longer double-limb support time than those without an exoskeleton. The variations in temporospatial parameters may be attributed to different mechanical designed exoskeletons. Regarding to this study, walking with either the default or intelligent-mode exoskeleton significantly decelerated velocity and cadence when compared to normal walking at both speed (p<0.001). Moreover, the default setting compromised the percentage of left swing but increased step width in contrast to walking without the exoskeleton.”

To

“The exoskeleton specifically changed gait parameters and walking habits in order to maintain the dynamic balance of the human body [30-32]. In many commercialized exoskeletons such as ReWalk, Ekso, etc., they usually adopt the basic joint trajectory tracking control as their default control [11]. The adaptive control, as in our case, is considered to be more advanced and unique for a wearer. Besides, compared to an exoskeleton from Cyberdyne using muscle signal as a trigger, its main purpose aims to create a gait movement with a body-supported device rather than solely assist a wearer energetically as for our Exo-H3 exoskeleton. Regarding to this study, walking with either the default or intelligent-mode exoskeleton significantly decelerated velocity and cadence when compared to normal walking at both speed (p<0.001). Moreover, the default setting compromised the percentage of left swing but increased step width in contrast to walking without the exoskeleton. Average baseline gait parameters demonstrated velocity 100 cm/s, stride length 1.2 m, and stance phase 61%. The applying exoskeleton resulted in 58-75 cm/s velocity reduction, 0.3-29 cm shorter stride length, and 4-10% longer stance phase. Despite temporospatial parameter variation attributed to different mechanical designed exoskeletons [33-36], age and neuromuscular involvement of participants [37, 38], the amounts of gait parameter changes in this study were comparable with the other multi-joint adaptive technology. In work by Swank, et al.[39], the robotic exoskeleton (EKSO) had a slower walking speed, shorter stride length, and longer double-limb support time than those without an exoskeleton. Comparing with and without Ekso in 15 health adults, average gait parameters showed velocity 31.0 ± 0.4 vs 132.0 ± 1.6 cm/s, stride length 0.72 ± 0.14 vs 1.41 ± 0.12 m, and double support 4.5 ± 0.6 vs 1.7 ± 0.2 s [39]”

**9. References: The following references are highly recommended to added to the paper to**

**improve its quality and state of the arts. (10.1109/TNSRE.2021.3136088),**

**(10.1109/ACCESS.2023.3325211), and (10.3390/s23136103).**

**Answer:**

Thank you so much for suggesting the valuable paper about single leg exoskeleton in children with cerebral palsy. We added these two references in the discussion part.

References

1. Reference 36: Sarajchi M, Al-Hares MK, Sirlantzis K. Wearable Lower-Limb Exoskeleton for Children With Cerebral Palsy: A Systematic Review of Mechanical Design, Actuation Type, Control Strategy, and Clinical Evaluation. IEEE Trans Neural Syst Rehabil Eng. 2021;29:2695-2720. doi: 10.1109/TNSRE.2021.3136088. Epub 2022 Jan 4. PMID: 34910636.
2. Reference 38: Sarajchi M, Sirlantzis K. Design and Control of a Single-Leg Exoskeleton with Gravity Compensation for Children with Unilateral Cerebral Palsy. Sensors (Basel). 2023 Jul 2;23(13):6103. doi: 10.3390/s23136103. PMID: 37447953; PMCID: PMC10346534.

**Revision:** Discussion, Line 423-425 was revised by adding these two important references.

From

“The variations in temporospatial parameters may be attributed to different mechanical designed exoskeletons.”

To

“Despite temporospatial parameter variation attributed to different mechanical designed exoskeletons [33-36], age and neuromuscular involvement of participants [37, 38]”

R**eviewer #2: As the statistical reviewer I will focus on methods and reporting.**

Major

**1) I struggled to follow the power calculations, or they are wrong. much more clarity is needed. if the means and SDs are reported and the authors calculated mean differences, i arrive at a very different sample calculation. Also it was not clear if an appropriate crossover design power calculation approach was used. This is highly problematic. also, even if this was correct for the primary outcome, it is extremely unlikely that power is adequate for other outcomes which is a major limitation.**

**Answer:**

Thank you for your comments regarding the power calculations. We apologize for any confusion caused and would like to clarify our approach. The sample size calculation for the crossover design was based on the guidelines provided by Assoc. Prof. Cameron Hurst from Chulalongkorn University. We used the sample size calculation tool available at this website <https://www2.ccrb.cuhk.edu.hk/stat/mean/tsmc_sup.htm>

After filling the required parameters specifically, a mean difference of 6.8 degrees (as cited in Guzik et al., 2020), a standard deviation (SD) of 7.6 degrees, and a margin of 5 degrees—the calculated total sample size was 8 participants for the crossover design. This calculation is illustrated in the figure below.

Reference

Guzik A, Drużbicki M, Wolan-Nieroda A, Turolla A, Kiper P. Estimating Minimal Clinically Important Differences for Knee Range of Motion after Stroke. J Clin Med. 2020 Oct 15;9(10):3305. doi: 10.3390/jcm9103305. PMID: 33076214; PMCID: PMC7602397.

**Revision:** I have revised the content of sample size calculation in the manuscript line 228-232

From

“Sample size estimation was performed based on evidence from the mean and standard deviations of peak value of knee extension of the unaffected leg in stroke patients with Knee Ankle Foot Orthosis (KAFO) (-0.8±7.6 degrees) compared to those without KAFO (0.2±7.2 degrees). The study used a type I error (alpha) of 0.05 and power of 0.8. Therefore, a sample size was 4 participants per group.”

To

“Sample size estimation was conducted using a sample size calculator for crossover design (https://www2.ccrb.cuhk.edu.hk/ stat/mean/tsmc_sup.htm). The estimation was based on a mean difference of 6.8 degrees [29], a standard deviation (SD) of 7.6 degrees, and a margin of 5 degrees. The study used a type I error (alpha) of 0.05 and power of 0.8. Therefore, the total sample size required for the study was 8 participants.”

**Revision:** I have revised the added the information in limitation part line 476-479

From

“The comfort and performance scores were modified from the previous studies, and results may apply with cautions because of this unique exoskeletal setting, including only healthy adults with forearm crutches assistance, and very small number of sample size.”

To

“The comfort and performance scores were modified from the previous studies, and results may apply with cautions because of this unique exoskeletal setting, including only healthy adults with forearm crutches assistance, and very small number of sample size contributed to major limitation.”

**2) how did the authors deal with missing data, can they expand to clarify in the methods section as per the CONSORT statement?**

**Answer:** We performed the real time data collection. Therefore, there was no missing data.

Minor

**1) Can this sentence be rephrased for clarity and grammer please: "The registration was delayed due to a misunderstanding between the project and the principal investigator resigned from the organization**

**Answer:** Thank you for your comment. I have revised the content in the manuscript line 105-106.

**Revision:**

From

“The trial protocol was retrospectively registered with clinicaltrials.gov identifier: NCT06513390. The registration was delayed due to a misunderstanding between the project and the principal investigator resigned from the organization.”

To

“The trial protocol was retrospectively registered with clinicaltrials.gov identifier: NCT06513390”

**2) Stata not STATA (not an acronym).**

**Answer:** Thank you for your comment**.** I have revised the content in the manuscript line 239-240.

**Revision:**

From

“Statistical analysis was performed using STATA software version 15.0”

To

“Statistical analysis was performed using Stata software version 15.0”

**Reviewer #3:** This paper compares between adaptative and non-adaptative control laws for robotic exoskeletons used in rehabilitation of lower limbs, using different metrics. Authors pointed out the interest in the CPG-based controllers, its robustness to perturbations, compared to other control methods.

By and large, the idea presented in this paper is attractive. However, several comments are raised and should be well addressed to improve this paper:

**1- The introduction is very short (compared to the abstract) and should be clearly expanded. Moreover, in introduction, it is not enough to state the current work. It should be expended and reconstructed. Including the motivation, the main difficulties, the main work and the improvements compared with previous related works should be emphasized in this section.**

**Answer:**

Thank you so much for your recommendation. Details of the original works of recent exoskeleton and its performance were elaborated. Also tracking error and interactive torque improvement was emphasized. This newly adaptive modular neural control interacts interpretable neural components to feedback signals. Its performances effectively compromised tracking errors (80%), and interaction torque (30%) when compared to the benchmark of the coupled cooperative primitives (CCPs) (Srisuchinnawong A 2023). Nevertheless, gait cycle learning and user interface in different speeds was still needed to be explored.

Reference

Srisuchinnawong A, Akkawutvanich C, Manoonpong P. "Adaptive Modular Neural Control for Online Gait Synchronization and Adaptation of an Assistive Lower-Limb Exoskeleton," in IEEE Transactions on Neural Networks and Learning Systems, doi: 10.1109/TNNLS.2023.3263044.

**Revision:** The manuscript has rewritten, line 47-95

From

“Medical assistive devices have been variously evolved to improve human moving abilities. Regarded as empowering technology, exoskeletons or robot suits have complemented walking and climbing support for many decades[1]. These appliances also extend, substitute, and enhance function, as well as play an important role in the treatment and rehabilitation of movement disorders[2]. Several studies found the lower-limb exoskeleton training was generally safe and feasible for the patient with gait problem such as spinal cord injury, stroke, cerebral palsy[3-8]. The effectiveness of exoskeleton training depends on individual factors such as the smoothness of its movement and coordination. Current device controllers mostly predefined joint trajectories without real-time online adaptation. The force interaction controllers or offline control-parameter optimization of exoskeletons was required to synchronize the lower limb movement[9]. Based on different environment conditions, the model-based, hierarchy-based, physical parameters-based, and usage-based control exoskeletal systems have been unfulfilled continuous movement, and energy-efficient gaits[9]. An alternative biologically-inspired control system based on central pattern generators (CPGs) can generate oscillatory movement and facilitate a natural walk along with a robust rhythmic gait pattern. Despite widespread adoption of CPGs in recent years [10-13], a comprehensive evaluation of their effectiveness is lacking.

This study addresses this gap by testing a state-of-the-art adaptive biologically-inspired control system based on CPGs (intelligent mode) [13] and comparing its performance to a standard commercial system (default mode) across multiple metrics, including three-dimensional gait analysis, electromyography, oxygen consumption, user comfort, and exoskeleton performance scores. The powered, mobile, lower-limb exoskeleton Exo-H3 by Technaid, Spain was employed for the investigation. The adaptive CPG-based control system utilized six neural control modules with online adaptation based on joint position tracking error and interaction torque [13]. In contrast, the basic control system utilized a predefined pattern gait with manually adjustable swing speed (frequency) but lacked real-time online adaptation during locomotion. Expected benefits of this adaptive version would ameliorate natural walking and essential lower limb motions.”

To

“Medical assistive devices have been variously evolved to improve human moving abilities. Regarded as empowering technology, exoskeletons or robot suits have complemented walking and climbing support for many decades [1]. These appliances also extend, substitute, and enhance function, as well as play an important role in the treatment and rehabilitation of movement disorders [2]. Several studies found the lower-limb exoskeleton training was generally safe and feasible for the patient with gait problem such as spinal cord injury, stroke, cerebral palsy [3-8].

The effectiveness of exoskeleton training depends on individual factors such as the smoothness of its movement and coordination. Thus, many studies [9] were focusing not only on hardware design, but also a development of efficient control algorithm. Generally, the control strategies can be classified into high-, mid-, or low-level control layers[10]. The high-level layer provides operation mode to the crucial mid-level layer where gait detection and synchronization occur. A basic control method depends on predefined joint trajectories and initiates different walking pattern by a user[11]. To increase adaptability in different conditions, some controls implemented gait planning based on a kinematic model[12] or a simplified nonlinear dynamic model as inverted pendulum[13]. The generated profiles were then controlled by different real-time trajectory tracking methods such as proportional-integral-derivative (PID) control integrated in the low-level motor driver or linear quadratic regulator (LQR) control. Advanced control strategies were introduced such as PID nonlinear controller with Linear Matrix Inequalities[14], backstepping control technique[15], and sliding mode control[16], to guarantee stability and robustness. However, those model-based methods required solving complex equations during the offline process. Apart from joint profile, there was torque related control scheme[17]. Nevertheless, it required a torque sensor for feedback to gain more accurate control over several situations.

Strictly to joint angle sensor, adaptive-oscillator-based or central pattern generator (CPG)-based control, which was considered as a biologically-inspired model-free approach, was introduced to handle rhythmic movement as walking. Its intrinsic property as limit cycle provided a stable joint trajectory and disturbance rejection by nature. Adaptive frequency oscillator (AFO) [18] was proposed to entrain frequency from feedback. It required fewer trails (~ six gait cycles) to adapt for individual user. Another recent adaptive trajectory/shape generation framework was dynamic movement primitive and hierarchical interactive learning (DMP-HIL) [19]. It tried to solve a synchronization problem by modeling as leader-follower agents. The method required approximately 15 gait cycles to converge. More techniques had been added to the aforementioned framework and turned it into coupled cooperative primitive (CCP) framework [20], which coupled the interaction model in both the velocity and acceleration levels. It has also been proven to outperform the conventional DMP-HIP in term of interaction torque reduction. However, the method could still not adapt to different frequencies.

To achieve online frequency and shape adaptations in response to a joint angle tracking error, our study[21] modified the previous CCP framework with CPG module called SO(2) oscillator[22] whose inputs were associated with gradients of the joint angle tracking error. This novel control was called an adaptive modular neural control (AMNC) with less adaptation time (within a gait cycle). Although, the proposed method for online gait synchronization effectively optimized the tracking error by 80% and interaction torque by 30% [21], a comprehensive evaluation of its effectiveness in term of spatiotemporal and biological signals on practical situation in real clinical environment has not been demonstrated before. This study addresses this gap by testing a state-of-the-art adaptive biologically-inspired control system based on CPGs (intelligent mode)[21], and comparing its performance to a standard commercial system (default mode) across multiple metrics, including three-dimensional gait analysis, electromyography, oxygen consumption, user comfort, and exoskeleton performance scores. Expected benefits of this adaptive version would ameliorate natural walking and essential lower limb motions.”

**2- The authors should polish the paper suitably. The whole paper should be reviewed carefully, in order to correct all the typing errors.**

**Answer:** Thank you for your feedback. We have carefully reviewed the entire manuscript and corrected all typing errors as suggested.

**3- The authors should give a Remark to illustrate how the design parameters effect the control performance of the exoskeleton in low and high speeds, and how to choose these parameters.**

**Answer:**

Thank you so much for your question and suggestion. The design parameters affected the control performance in both speeds were detailed and illustrated in the supplementary file (S1 File). The parameters were the same for both speeds. Only 10% incremental of the preferred speed was set as the high speed.

In this study, we changed only speed, but the other parameters were the same for both levels. The set of weights used to generate joint trajectories (hip, knee, and ankle) are the same for both speeds. The pattern generator block has learnt the weights from the default patterns provided by the manufacturer of Exo-H3 during the offline process. The same pattern is applied for both speeds during the default mode also. This might result in slightly different patterns which are still acceptable for guiding the subjects.

For phase generator where CPG is deployed, all parameters are tuned to be able to generate certain swing frequency range (0 < swing freq. < 1 Hz). This range covers all low and high speeds in our experiments.

**Revision:** The manuscript has revised by adding designed parameters’ details in the intervention part line 148-160

From

“Full descriptions of Exo-H3 hardware and the development of default and intelligent modes are available in the supplementary material (S1 File). Note that the Exo-H3 functions as an adaptive exoskeleton when using the intelligent control mode, and as a non-adaptive exoskeleton with the default mode.”

To

“Full descriptions of Exo-H3 hardware and the development of default and intelligent modes are available in the supplementary material (S1 File). Note that the Exo-H3 functions as an adaptive exoskeleton when using the intelligent control mode, and as a non-adaptive exoskeleton with the default mode. The design parameters affected the control performance in both speeds were detailed and illustrated in the supplementary file (S1 File). The parameters were the same for both speeds. Only 10% incremental of the preferred speed was set as the high speed. The set of weights used to generate joint trajectories (hip, knee, and ankle) are the same for both speeds. The pattern generator block has learnt the weights from the default patterns provided by the manufacturer of Exo-H3 during the offline process. The same pattern was also applied for both speeds during the default mode. This might result in slightly different patterns which were still acceptable for guiding the subjects. For phase generator where CPG is deployed, all parameters were tuned to be able to generate certain swing frequency range (0 < swing freq. < 1 Hz). This range covered all low and high speeds in our experiments.”

**4- As a clinical trial study, how you quantity the novelty of the proposed method ?**

**Answer:**

Thank you so much for your question. The novelty of this proposed method was quantified by percentage of tracking error and interaction torque optimization when compared to the default mode. From the previous research (Srisuchinnawong A 2023), tracking error was reduced by 80%, and obscured interaction torque was omitted by 30%. Proposed novel strategy that solely optimizes the positional tracking error of an assistive lower-limb exoskeleton, enabling the exoskeleton to automatically synchronize the walking phase and trajectory shape, thereby reducing the average interaction torque. When comparing the interaction torques of the AMNC and CCP control, the reduction in tracking error leads to a decrease of 7%–30% in the hip joints, 10%–30% in the knee joints, and 3%–13% in the ankle joints (p-value < 0.05, Mann–Whitney U test).

**Revision:** The manuscript has rewritten in the Introduction part line 83-95

From

“This study addresses this gap by testing a state-of-the-art adaptive biologically-inspired control system based on CPGs (intelligent mode) [13] and comparing its performance to a standard commercial system (default mode) across multiple metrics, including three-dimensional gait analysis, electromyography, oxygen consumption, user comfort, and exoskeleton performance scores. The powered, mobile, lower-limb exoskeleton Exo-H3 by Technaid, Spain was employed for the investigation. The adaptive CPG-based control system utilized six neural control modules with online adaptation based on joint position tracking error and interaction torque [13]. In contrast, the basic control system utilized a predefined pattern gait with manually adjustable swing speed (frequency) but lacked real-time online adaptation during locomotion. Expected benefits of this adaptive version would ameliorate natural walking and essential lower limb motions.”

To

“To achieve online frequency and shape adaptations in response to a joint angle tracking error, our study [13] modified the previous CCP framework with CPG module called SO(2) oscillator [14] whose inputs were associated with gradients of the joint angle tracking error. This novel control was called an adaptive modular neural control (AMNC) with less adaptation time (within a gait cycle). Although, the proposed method for online gait synchronization effectively optimized the tracking error by 80% and interaction torque by 30% [13], a comprehensive evaluation of its effectiveness in term of spatiotemporal and biological signals on practical situation in real clinical environment has not been demonstrated before. This study addresses this gap by testing a state-of-the-art adaptive biologically-inspired control system based on CPGs (intelligent mode) [13], and comparing its performance to a standard commercial system (default mode) across multiple metrics, including three-dimensional gait analysis, electromyography, oxygen consumption, user comfort, and exoskeleton performance scores. Expected benefits of this adaptive version would ameliorate natural walking and essential lower limb motions”

**5- Related to previous question, there are some control methods that are based on SMC and also those based on linear matrix inequality to design gains and improve performances according to some gains. These SMC- and LMI-based two control methods have been considered recently for lower limber exoskeletons. I suggest to see for example the following papers “An LMI-based robust state-feedback controller design for the position control of a knee rehabilitation exoskeleton robot: Comparative analysis”, “Fast terminal sliding mode control with rapid reaching law for a pediatric gait exoskeleton system”, “Adaptive backstepping sliding mode subject-cooperative control for a pediatric lower-limb exoskeleton robot”, “Design and Validation of a Pediatric Gait Assistance Exoskeleton System with Fast Non-Singular Terminal Sliding Mode Controller”**

**Answer:**

Thank you so much for your suggestion. Regarding to your suggested articles, the Linear Matrix Inequalities (LMIs) was formulated for the feedback gain of the proposed controller and stabilizer of the knee exoskeleton utilizing the Lyapunov technique (Jenhani S, 2024). Also, an improved fast non-singular terminal sliding mode (IFNSTSM) provided superb control performance when compared to fast non-singular terminal sliding mode (FNSTSM) control and timescaling sliding mode (TSSM) control (Narayan J 2024).

References

1. Jenhani S, Gritli H. An LMI-based robust state-feedback controller design for the position control of a knee rehabilitation exoskeleton robot: Comparative analysis. Measurement and Control. 2024;0(0). doi:10.1177/00202940241236295
2. Narayan J, Abbas M, Dwivedy SK. Design and validation of a pediatric gait assistance exoskeleton system with fast non-singular terminal sliding mode controller. Med Eng Phys. 2024 Jan;123:104080. doi: 10.1016/j.medengphy.2023.104080. Epub 2023 Dec 7. PMID: 38365333.

**Revision:** We have cited some papers about LMI-based, and IFNSTSM as well as adaptive backstepping in the introduction

From

“Medical assistive devices have been variously evolved to improve human moving abilities. Regarded as empowering technology, exoskeletons or robot suits have complemented walking and climbing support for many decades[1]. These appliances also extend, substitute, and enhance function, as well as play an important role in the treatment and rehabilitation of movement disorders[2]. Several studies found the lower-limb exoskeleton training was generally safe and feasible for the patient with gait problem such as spinal cord injury, stroke, cerebral palsy[3-8].

The effectiveness of exoskeleton training depends on individual factors such as the smoothness of its movement and coordination. Current device controllers mostly predefined joint trajectories without real-time online adaptation. The force interaction controllers or offline control-parameter optimization of exoskeletons was required to synchronize the lower limb movement[9]. Based on different environment conditions, the model-based, hierarchy-based, physical parameters-based, and usage-based control exoskeletal systems have been unfulfilled continuous movement, and energy-efficient gaits[9]. An alternative biologically-inspired control system based on central pattern generators (CPGs) can generate oscillatory movement and facilitate a natural walk along with a robust rhythmic gait pattern. Despite widespread adoption of CPGs in recent years [10-13], a comprehensive evaluation of their effectiveness is lacking.”

To

“Medical assistive devices have been variously evolved to improve human moving abilities. Regarded as empowering technology, exoskeletons or robot suits have complemented walking and climbing support for many decades [1]. These appliances also extend, substitute, and enhance function, as well as play an important role in the treatment and rehabilitation of movement disorders [2]. Several studies found the lower-limb exoskeleton training was generally safe and feasible for the patient with gait problem such as spinal cord injury, stroke, cerebral palsy [3-8]. The effectiveness of exoskeleton training depends on individual factors such as the smoothness of its movement and coordination. Thus, many studies [9] were focusing not only on hardware design, but also a development of efficient control algorithm. Generally, the control strategies can be classified into high-, mid-, or low-level control layers[10]. The high-level layer provides operation mode to the crucial mid-level layer where gait detection and synchronization occur. A basic control method depends on predefined joint trajectories and initiates different walking pattern by a user[11]. To increase adaptability in different conditions, some controls implemented gait planning based on a kinematic model[12] or a simplified nonlinear dynamic model as inverted pendulum[13]. The generated profiles were then controlled by different real-time trajectory tracking methods such as proportional-integral-derivative (PID) control integrated in the low-level motor driver or linear quadratic regulator (LQR) control. Advanced control strategies were introduced such as PID nonlinear controller with Linear Matrix Inequalities[14], backstepping control technique[15], and sliding mode control[16], to guarantee stability and robustness. However, those model-based methods required solving complex equations during the offline process. Apart from joint profile, there was torque related control scheme[17]. Nevertheless, it required a torque sensor for feedback to gain more accurate control over several situations.”

**6- There are several figures included in the results. It is good, but it is difficult to read them easily. Then, I suggest to add Tables to recapitulate the necessary and important details from these figures**

**Answer:**

Thank you for your suggestion. According to reviewer comment we summarize the important information from figures3-10 in Table 3. Figures 3–6 show the comparisons of mean pelvic hip, knee, and ankle kinematic angles of the exoskeleton mode with normal walking, as well as the intelligent mode with the exoskeleton's default mode at both normal and high speeds. Likewise, Figures 7–10 present the SPM paired t-test results for the comparisons of pelvic hip, knee, and ankle angles of the exoskeleton mode versus normal walking and intelligent mode versus default mode of the exoskeleton at normal and high speed.
